# Notch signaling regulates UNC5B to suppress endothelial proliferation, migration, junction activity, and retinal plexus branching

**DOI:** 10.1038/s41598-024-64375-z

**Published:** 2024-06-13

**Authors:** Qanber Raza, Taliha Nadeem, Seock-Won Youn, Bhairavi Swaminathan, Ahana Gupta, Timothy Sargis, Jing Du, Henar Cuervo, Anne Eichmann, Susan L. Ackerman, L. A. Naiche, Jan Kitajewski

**Affiliations:** 1 Department of Physiology and Biophysics, College of Medicine, University of Illinois at Chicago, 1853 W Polk St, Rm 522 (MC 901), Chicago, IL 60612 USA; 2 Centro Nacional de Investigaciones Cardiovasculares Carlos III- CNIC- (F.S.P), Madrid, Spain; 3 Yale School of Medicine, New Haven, CT USA; 4 University of San Diego, San Diego, CA USA; 5 University of Illinois Cancer Center, Chicago, USA

**Keywords:** Retinal angiogenesis, Notch effectors, UNC5B, Endothelial proliferation, Endothelial migration, Cell–cell adhesion, Angiogenesis, Cell signalling, Gene regulation, Cell polarity

## Abstract

Notch signaling guides vascular development and function by regulating diverse endothelial cell behaviors, including migration, proliferation, vascular density, endothelial junctions, and polarization in response to flow. Notch proteins form transcriptional activation complexes that regulate endothelial gene expression, but few of the downstream effectors that enable these phenotypic changes have been characterized in endothelial cells, limiting our understanding of vascular Notch activities. Using an unbiased screen of translated mRNA rapidly regulated by Notch signaling, we identified novel in vivo targets of Notch signaling in neonatal mouse brain endothelium, including *UNC5B*, a member of the netrin family of angiogenic-regulatory receptors. Endothelial Notch signaling rapidly upregulates UNC5B in multiple endothelial cell types. Loss or gain of UNC5B recapitulated specific Notch-regulated phenotypes. UNC5B expression inhibited endothelial migration and proliferation and was required for stabilization of endothelial junctions in response to shear stress. Loss of UNC5B partially or wholly blocked the ability of Notch activation to regulate these endothelial cell behaviors. In the developing mouse retina, endothelial-specific loss of UNC5B led to excessive vascularization, including increased vascular outgrowth, density, and branchpoint count. These data indicate that Notch signaling upregulates UNC5B as an effector protein to control specific endothelial cell behaviors and inhibit angiogenic growth.

## Introduction

Notch signaling is an essential regulator of developmental and pathological angiogenesis. Abnormally high or low Notch signaling has deleterious effects on angiogenesis and frequently contributes to the pathogenesis of angiogenesis-dependent diseases such as diabetic retinopathy, cardiovascular diseases, and cancer^1–5^.

Notch proteins are translated as a single polypeptide which is cleaved at an S1 cleavage site adjacent to the transmembrane domain, and the resulting extracellular domain is associated with the transmembrane and intracellular domains via cysteine disulfide bonds^6^. Ligand interaction with the extracellular domain generates conformational changes that trigger S3 cleavage by gamma (γ)-secretase, which releases the intracellular domain (ICD) to the cytosol^7–10^. The ICD translocates to the nucleus, where it binds to the transcriptional co-factor RBPJ (also known as CSL or CBF1) and co-activator MAML to promote the transcription of target genes that impact critical cellular functions^11,12^. This proteolytic cascade can be blocked in experimental or clinical settings using γ-secretase inhibitors (GSIs)^13–15^. Notch signaling can be activated by breaking the disulfide bonds using chelating agents such as EGTA, which releases the extracellular domain to allow rapid γ-secretase cleavage^16–18^. Similarly, expression of truncated Notch proteins with minimal or no extracellular domains results in rapid γ-secretase cleavage and activation of transcription and is an important driving force in multiple forms of cancer^19–21^. Of the four mammalian Notch receptors, endothelial cells primarily express Notch1 and Notch4^22,23^.

In endothelial cells, Notch signaling regulates sprouting angiogenesis and arterial specification^24^. Notch signaling limits sprouting angiogenesis by regulating the formation of endothelial tip cells^25^. Notch ligand Delta-like 4 (DLL4) in the leading endothelial tip cell activates Notch1 in neighboring endothelial cells (stalk cells) to suppress tip cell fate and promote stalk cell phenotypes, including reduced migratory activity and filopodial activity, and increased endothelial cell–cell junction stability^26^. Suppression of Notch signaling in primary endothelial cells or in developing vasculature confers a more tip-like phenotype, including increased migration and disrupted endothelial adherens junctions^27^.

Notch signaling regulates the emergence of arterial endothelial cells by controlling endothelial proliferation and cell cycle arrest^28,29^. In cultured primary endothelial cells, Notch1 signaling restricts cell proliferation^29^. In the retinal vascular plexus, Notch signaling triggers cell cycle arrest, which is sufficient to bias endothelial cells towards an arterial fate^28^. Endothelial-specific loss of transcriptional co-factor *Rbpj* leads to increased endothelial cell proliferation in the retina^30^. Retinal vasculature with mosaic Notch gain- and loss-of-function revealed that either excessive or absent Notch signaling blocked endothelial proliferation and arterial fate^31^. Once arteries have formed, laminar flow maintains ongoing Notch signaling, which is required to orient endothelial cells in the direction of flow and form stable endothelial junctions^29^.

Despite the wealth of published information regarding phenotypes regulated by endothelial Notch signaling, few Notch targets that enact these phenotypes are known or understood. Well-characterized genes activated by the Notch transcriptional complex include members of the Hairy/Enhancer of Split (*Hes*) and Hair/Enhancer of Split related to YRPW motif (*Hey*) families^32–35^, which act as negative regulators of Notch signaling^32,35^. Other direct targets include VEGFR3 (*Flt4*), which modulates vascular permeability^36,37^, ephrin B2 (*Efnb2*), a potent regulator of endothelial migration and proliferation^38,39^, and the Notch regulated ankyrin repeat protein (*Nrarp*), which regulates vascular density^40,41^. Hey family member *Hey2* is a direct suppressor of *VEGFR2* (also known as *Kdr*), an essential receptor of vascular endothelial growth factor A (VEGF-A), a powerful chemoattractant which triggers endothelial migration, proliferation, and sprouting^42,43^. Identification of a more complete set of the angiogenic genes regulated by endothelial Notch signaling is critical for better understanding of endothelial function and more precisely targeted interventions in angiogenesis-dependent diseases.

To identify novel mediators of Notch angiogenic function in vivo, we screened differentially expressed genes in postnatal day 8 (P8) mice treated with a GSI to inhibit Notch signaling. To enrich for direct Notch targets, we focused on genes that respond rapidly to the loss of Notch signaling. We identified novel genes not previously known to interact with endothelial Notch signaling, including *UNC5B*, a member of the netrin family of receptors^44,45^. UNC5B has previously been identified as a critical mediator of vascular development that shares many characteristics with Notch signaling, including enriched expression in arteries and hypervascularization phenotypes when suppressed by genetic knockouts or neutralizing antibodies^45–47^. UNC5B has also been implicated in pathological angiogenesis of retinal neovascularization and in the regulation of the blood–brain barrier integrity^48,49^.

Here, we demonstrate that UNC5B is rapidly regulated in endothelial cells in response to Notch signaling and that loss of UNC5B recapitulates Notch loss-of-function phenotypes such as increased endothelial proliferation, migration, and hypersprouting and decreased ability to polarize in response to laminar flow. Loss of UNC5B blocks the phenotypic effects of Notch activation, indicating that it is required downstream of Notch signaling. These results suggest that UNC5B is a critical effector of Notch signaling in angiogenesis.

## Results

### Loss of Notch signaling rapidly downregulates a novel transcriptional profile

**Figure 1:**
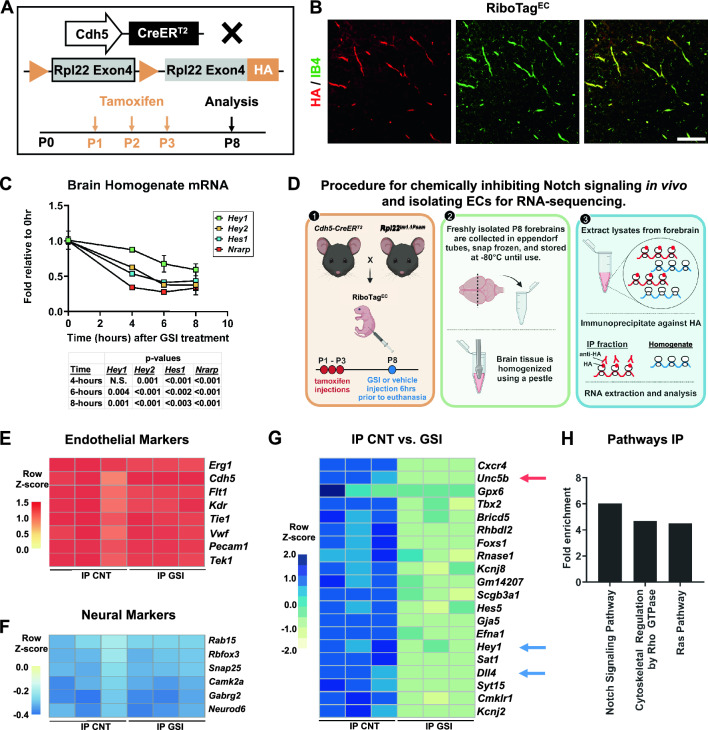
Characterization of rapidly regulated endothelial Notch targets in the developing mouse brain. (**A**) Diagram of the tamoxifen administration timeline and RiboTag^EC^ mouse harvest at postnatal day (P) 8. (**B**) Validation of RiboTag recombination efficiency and specificity in brain endothelium. P8 RiboTag^EC^ forebrain sections stained for vasculature with Isolectin B4 (IB4, green) and tagged ribosomes (anti-HA, red). Scale bar, 115 μm. (**C**) Time course of suppression of previously characterized canonical Notch target genes *Hey1*, *Hey2*, *Hes1*, and *Nrarp* after GSI treatment. (**D**) Diagram of the experimental workflow of RiboTag-based isolation of immunoprecipitated (IP) endothelial ribosomes and bulk brain homogenate for RNA extraction and analysis. (**E**,**F**) Heatmaps of RNAseq analysis of IP compared to forebrain homogenate. The IP fraction is highly enriched in endothelial markers (**E**) and depleted of neural markers (**F**). (**G**) Heatmap of the 20 most significantly downregulated genes in the GSI-treated brain endothelium. Known Notch targets *Hey1* and *Dll4* (blue arrows) and novel target *Unc5B* (red arrow) were significantly suppressed in GSI-treated animals. (**H**) GSEA analysis of pathways enriched in the IP endothelial fraction. All heatmaps indicate a z-score.

Little is known about the primary transcriptional events of Notch activation in endothelial cells that facilitate vascular development and function. To gain insight into dynamic changes in endothelial gene expression regulated by Notch signaling during angiogenesis, we used *Rpl22*^*tm1.1Psam*^ RiboTag mice^50^. RiboTag permits Cre-dependent expression of HA-tagged ribosomal protein (Rpl22) exon 4, labeling the ribosomes of Cre-expressing cells. This labeling enables direct immunoprecipitation (IP) of cell type-specific ribosomes from tissue homogenate, permitting isolation of translated endothelial mRNA without disrupting Notch signaling via tissue disaggregation. *Rpl22*^*tm1.1Psam*^ mice were bred to tamoxifen-inducible and endothelial cell-specific *Cdh5(PAC)-CreER*^*T2*^ (*Cdh5-CreER*^*T2*^) transgenic mice^51^ to create endothelial cell-specific ribosome labeling (RiboTag^EC^). To induce RiboTag recombination, we administered tamoxifen to RiboTag^EC^ mice (100 µg/pup/day) at postnatal days (P) 1–3 (Fig. 1A). Expression of HA-tagged Rpl22 protein was detected throughout the vascular endothelium of RiboTag^EC^ mouse brains at P8 and HA detection was not observed in non-endothelial cell types (Fig. 1B). To suppress Notch signaling, we treated mice with the γ-secretase inhibitor (GSI) DAPT^52^ (100 mg/kg, subcutaneously) or vehicle control at P8, a timepoint at which the forebrain microvasculature is actively angiogenic^53^. Brains were harvested at 0, 4, 6, and 8 h after GSI treatment. Quantitative reverse transcriptase-PCR (RT-qPCR) analysis of homogenate confirmed the downregulation of well-known Notch direct targets, such as *Hey1*, *Hey2*, *Hes1*, and *Nrarp*. All targets except for *Hey1* were significantly downregulated by GSI 4 h after treatment, and by 6 h after treatment, all targets were significantly downregulated (Fig. 1C). We thus demonstrate that inhibition of endothelial Notch signaling can be seen in neonatal brain endothelium after 6 h of GSI treatment.

P8 endothelial RiboTag^EC^ mouse brains were harvested 6 h after GSI treatment and used to generate whole-tissue homogenate mRNA pools or subjected to anti-HA IP to isolate ribosome-bound endothelial specific transcripts (Fig. 1D). Homogenate and IP mRNA were subjected to sequence analysis. Principal component analysis of RNA-sequence data demonstrated clear segregation between homogenate and IP populations and treatment groups (Supplemental Fig. 1A). To test IP samples for selective purification of endothelial mRNA, we examined expression levels of known endothelial-specific genes (*Erg1, Cdh5, Flt1, Kdr, Tie1, Vwf, Pecam1,* and *Tek1*). The expression of endothelial-specific markers was significantly and consistently enriched by approximately 35-fold in the IP fraction compared to homogenate in all samples (Fig. 1E and Supplemental Fig. 1B). The IP fraction was significantly depleted of neural, astrocyte, oligodendrocyte, and microglial markers (Fig. 1F and Supplemental Fig. 1C). These analyses confirmed the endothelial specificity of the RiboTag-mediated IP isolation from brain homogenates.

To identify novel Notch targets in the brain endothelium, we interrogated RNA-seq data for downregulated mRNA in the GSI-treated RiboTag IP samples with a significance cutoff of adjusted *P* value < 0.05 and log_2_ fold change < -0.27 (i.e., -1.2 fold). GSI treatment significantly downregulated 1566 genes in brain endothelial cells (Accession number GSE163568). We observed downregulation of previously identified Notch targets, including *Hey1* and *Dll4* (Fig. 1G and Supplemental Fig. 1D). Statistical over-representation analysis of the IP fraction via PANTHER GO confirmed that the Notch signaling pathway was downregulated and identified changes in pathways regulating motility and cytoskeleton (Fig. 1H). Additional analysis of this data has been discussed elsewhere^52^.

A strongly and significantly regulated novel candidate Notch target was *Unc5B*, a member of the netrin family of receptors (Fig. 1G and Supplemental Fig. 1D). Previous studies using neutralizing antibodies or global knockouts in mice and zebrafish showed that Unc5B controls vascular morphogenesis by negatively regulating capillary branching^45–48^, a phenotype similar to that seen with the reduction of endothelial Notch signaling. We thus *hypothesize* that Unc5B could act as an effector of Notch signaling to inhibit angiogenesis.

### UNC5B is rapidly regulated in response to Notch signal activation in cultured endothelial cells

**Figure 2:**
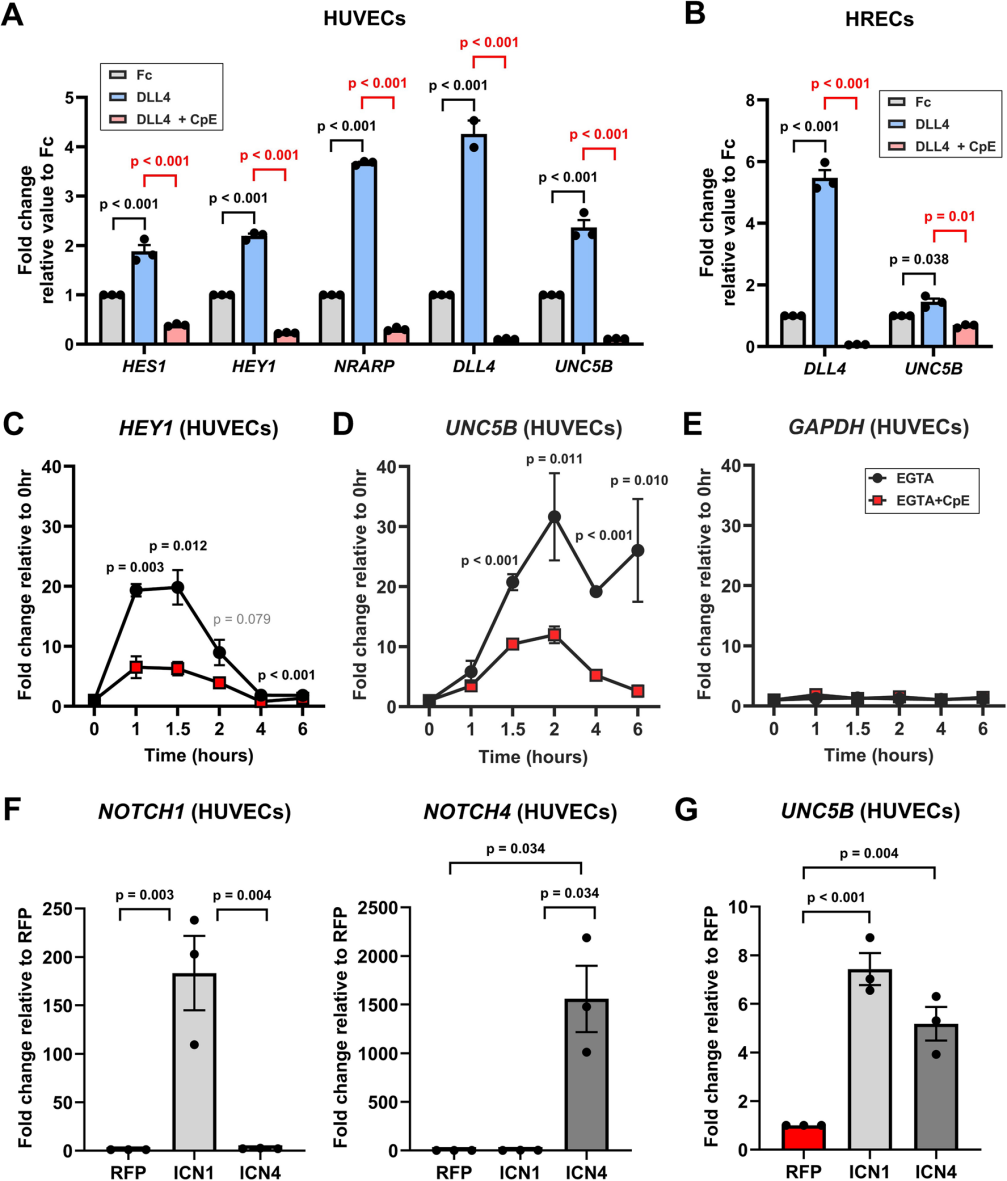
*UNC5B* is a novel target of Notch in endothelial cells. (**A**,**B**) Induction of Notch signaling in Human Umbilical Vein Endothelial Cells (HUVEC) using DLL4-Fc coated TLA plates significantly upregulate expression of Notch target genes *HES1, HEY1, NRARP,* and *DLL4* (blue bars). This effect is blocked by GSI treatment with Compound E (CpE, pink bars). *UNC5B* expression is significantly upregulated by Notch signaling and this upregulation is significantly blocked by CpE (right). Expression levels were evaluated by qPCR. (**C**–**E**) EGTA induction (black lines) and CPE inhibition of EGTA (red lines) of canonical Notch target *HEY1* (**C**), *UNC5B* (**D**), and *GAPDH* (**E**) in HUVECs. (F,**G**) HUVECs were lentivirally transduced with control RFP, ICN1, or ICN4 expression constructs. 24 h after lentivirus infection, cells were harvested and analyzed by qPCR for *NOTCH1*, *NOTCH4*, and *UNC5B* expression. Two-way ANOVA (**A**,**B**), multiple unpaired t-tests (**C**–**E**) and one-way ANOVA (**F**,**G**), presented as mean ± s.e.m. from at least 3 different biological replicates per experiment.

Notch ligands and receptors function on the cell surface of adjacent cells, and physical tension between the ligand and receptor is required for signal activation. To confirm that endothelial *UNC5B* is regulated by Notch signaling, we activated Notch signaling by immobilizing a ligand on tissue culture plates to permit force generation between the ligand and adjacent receptor expressing cells (tethered ligand assay, TLA). The extracellular domain of recombinant DLL4-Fc (10 μg/ml) or control Fc was immobilized on tissue culture plates and plates were seeded with Human Umbilical Vein endothelial cells (HUVECs) or Human Retinal Endothelial Cells (HRECs). Analysis of samples collected 6 h after seeding showed that DLL4 stimulation of Notch signaling caused significant induction of Notch canonical targets in both cell types, including *HES1*, *HEY1*, *NRARP*, and *DLL4* (Fig. 2A,B). DLL4 induction stimulated significant upregulation of *UNC5B* in HUVEC and HREC (Fig. 2A,B). To ensure that this upregulation was specific to activation of the Notch pathway, we treated HUVEC and HREC with 500 mM of the GSI Compound E (CpE). CpE significantly suppressed induction of canonical Notch targets and of *UNC5B* (Fig. 2A,B). These data indicate that activation of Notch signaling upregulates *UNC5B* in a γ-secretase dependent manner.

Direct transcriptional Notch targets are rapidly regulated by Notch activation^52^. To determine the kinetics of *UNC5B* regulation, we treated HUVECs or HRECs with EGTA, which disassociates the Notch extracellular domain and permits precise timing of Notch signal activation. A 15-min treatment with EGTA significantly induced both the canonical Notch target *HEY2* and *UNC5B* within 1–2 h in HUVEC and HREC (Supplemental Fig. 2A,B). This induction was significantly suppressed by CpE, confirming that EGTA regulates *UNC5B* via the Notch signaling pathway (Supplemental Fig. 2A,B). We further examined the kinetics of *UNC5B* regulation for 6 h after Notch activation by EGTA. In this longer timecourse, canonical Notch target *HEY1* was maximally upregulated (~ 20 fold) at 1–1.5 h and then declined thereafter (Fig. 2C). *UNC5B* was similarly upregulated 20–30 fold by 1.5 h and remained at that plateau for the length of the observed timecourse, suggesting that *UNC5B* either has a more stable transcript than *HEY1* or there are additional factors supporting its ongoing expression (Fig. 2D). Induction of both genes was suppressed by CpE (Fig. 2C,D). To confirm that gene expression was not globally altered by EGTA or CpE treatment, we examined expression of the non-Notch target *GAPDH*, which was not induced by EGTA nor suppressed by CpE (Fig. 2E). Based on these data, we conclude that endothelial UNC5B can be rapidly upregulated in response to Notch activation and is likely a direct target.

Of the Notch proteins, endothelial cells primarily express Notch1 and Notch4^22,23^. To examine the capacity of Notch1 and Notch4 to regulate *UNC5B,* we expressed constitutively active intracellular domains (ICDs) of human Notch1 (ICN1) and human Notch4 (ICN4) in endothelial cells to activate their respective signaling pathways. We validated significant upregulation of *NOTCH1* and *NOTCH4* ICDs by RT-qPCR 24 h after lentiviral transduction by ICN1, ICN4, or RFP control expression vectors (Fig. 2F). The ectopic expression of Notch1 ICD had minimal effect on the expression Notch4 and vice versa. To confirm activation of the Notch pathway by ICN1 and ICN4, we determined that each ICN protein upregulated canonical Notch targets *HES1*, *HEY1*, and *NRARP,* and a recently identified novel Notch target *RND1*^52^ (Supplemental Fig. 2C–F). In HUVECs, activation of Notch signaling by ICN1 and ICN4 proteins upregulated expression of *UNC5B* by 7.4-fold and 5.2-fold, respectively (Fig. 2G), suggesting that both Notch1 and Notch4 can regulate UNC5B transcription in endothelial cells.

The rapid response of *UNC5B* induction suggests that *UNC5B* is a direct Notch transcriptional target. Examination of the *UNC5B* genomic region in the ENCODE database indicated that there are two open chromatin DNAse hypersensitivity peaks near the *UNC5B* transcriptional start site. One of these open chromatin regions was observed only in endothelial cells, suggesting that this region encodes an endothelial-specific *UNC5B* promoter (Supplemental Fig. 3A). This region exhibited histone marks (H3k4m3) typical of promoters and contained two RBPJ-binding consensus sequences (Supplemental Fig. 3B,C). This suggests that this promoter may mediate direct Notch transcriptional regulation of *UNC5B* in endothelial cells.

### UNC5B regulates endothelial cell morphology, proliferation, and migration downstream of Notch signaling

**Figure 3:**
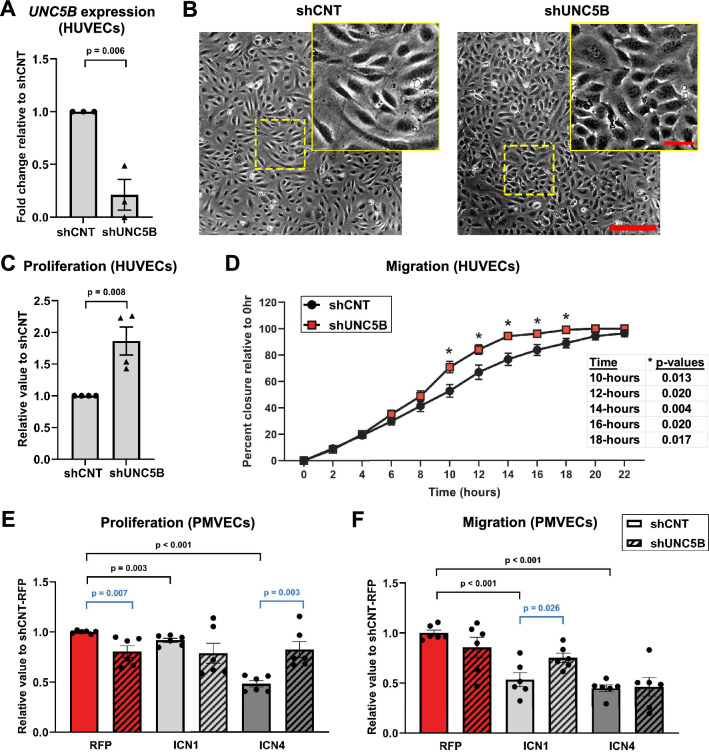
UNC5B regulates endothelial cell proliferation and migration downstream of Notch signaling. HUVECs were lentivirally transduced with scramble control (shCNT) or shRNA targeting UNC5B (shUNC5B). (**A**) qPCR for *UNC5B* expression 24 h after lentivirus infection. (B) Representative images of shCNT and shUNC5B HUVEC cell morphology. Yellow boxed inserts show zoomed images for cellular morphology. (**C**) shCNT and shUNC5B HUVEC proliferation measured by MTT assay. (**D**) Percent closure of scratch wounds in shCNT and shUNC5B HUVEC monolayers. Asterisks indicate the time points for significant differences in migration between shCNT and shUNC5B. (**E**) Proliferation of PMVECs transduced with ICN1 or ICN4 expression vectors and shCNT or shUNC5B, relative to shCNT-RFP control. (**F**) Cell migration values of PMVECs transduced with ICN1 or ICN4 expression vectors and shCNT or shUNC5B, relative to shCNT-RFP control. Unpaired t-tests (**A**,**C**) and multiple comparison unpaired t-tests (**D**–**F**), presented as mean ± s.e.m. Each dot represents an independent experiment with 4 replicates per experiment. Scale bars, 150 μm and 35 μm (zoomed).

Endothelial Notch signaling suppresses endothelial proliferation and migration, which drives stalk cell and arterial phenotypes^29,30,54,55^. We evaluated the effects of *UNC5B* knockdown on endothelial cellular function using shRNA targeted against *UNC5B* (shUNC5B) which reduced *UNC5B* transcript levels by approximately 85% and protein levels by approximately 67% (Fig. 3A, Supplemental Fig. 4A,B). Knockdown of *UNC5B* produced morphological changes in HUVEC monolayers, including an increased number of cells per visual field, suggesting an increase in proliferation (Fig. 3B). An MTT cell viability assay performed 72 h after transfection with shUNC5B and shCNT showed that HUVECs with *UNC5B* knockdown were more proliferative relative to shCNT HUVEC (Fig. 3C). To evaluate the effects of UNC5B on endothelial migration, we used a scratch wound-healing assay in which the extent of migration of cells into cleared area was examined. At intermediate stages of closure, the percentage closure of shUNC5B-treated HUVECs was significantly greater than that of shCNT-treated cells (Fig. 3D). Thus, UNC5B plays a role in suppressing endothelial proliferation and migration.

Endothelial cells from different vascular beds can have distinct regulation, and current recommendations suggest that in vitro studies should confirm findings in HUVECs with endothelial cells from a different, non-venous, vascular origin^56^. We therefore expanded our examination into pulmonary microvascular endothelial cells (PMVECs), where Notch signaling is moderately active under physiologic conditions and Notch4 in particular is expressed at high levels^57^. We transduced PMVECs with either shCNT or shUNC5B and confirmed *UNC5B* knockdown via qPCR (Supplemental Fig. 2G, red bars). In PMVEC, loss of *UNC5B* alone significantly reduced proliferation, confirming our results in HUVEC (Fig. 3E, red bars). Loss of *UNC5B* reduced migration to a non-significant extent in PMVEC, suggesting that this microvascular cell type required *UNC5B* for migration to a lesser extent under baseline conditions (Fig. 3F, red bars).

These data demonstrate that loss of *UNC5B* in endothelial cells recapitulates specific Notch loss-of-function phenotypes. To determine if *UNC5B* functions as an effector of the Notch pathway, we activated Notch signaling and then tested whether knockdown of *UNC5B* would block the effects of increased Notch signaling (Supplemental Fig. 2G, gray bars). As predicted, activation of the Notch pathway via overexpression of either ICN1 or ICN4 reduced proliferation in PMVEC cells (Fig. 3E, dark gray bars). ICN4 showed a much stronger degree of proliferative suppression, suggesting that Notch4 is a significant regulator of endothelial proliferation in lung endothelial cells. The knockdown of *UNC5B* significantly rescued endothelial proliferation in the presence of ICN4, indicating that *UNC5B* is required for Notch4-mediated suppression of endothelial proliferation (Fig. 3E, dark gray bars). Migration of PMVECs was significantly reduced by expression of either ICN1 or ICN4 (Fig. 3F, gray bars). The knockdown of *UNC5B* significantly rescued INC1-mediated migration suppression but had no significant effect on ICN4-mediated migration suppression (Fig. 3F, gray bars). Altogether, these findings suggest that *UNC5B* acts as a required Notch effector mediating endothelial migration and proliferation in specific cellular contexts.

### UNC5B regulates Notch-induced endothelial junction stability and cell polarization

**Figure 4:**
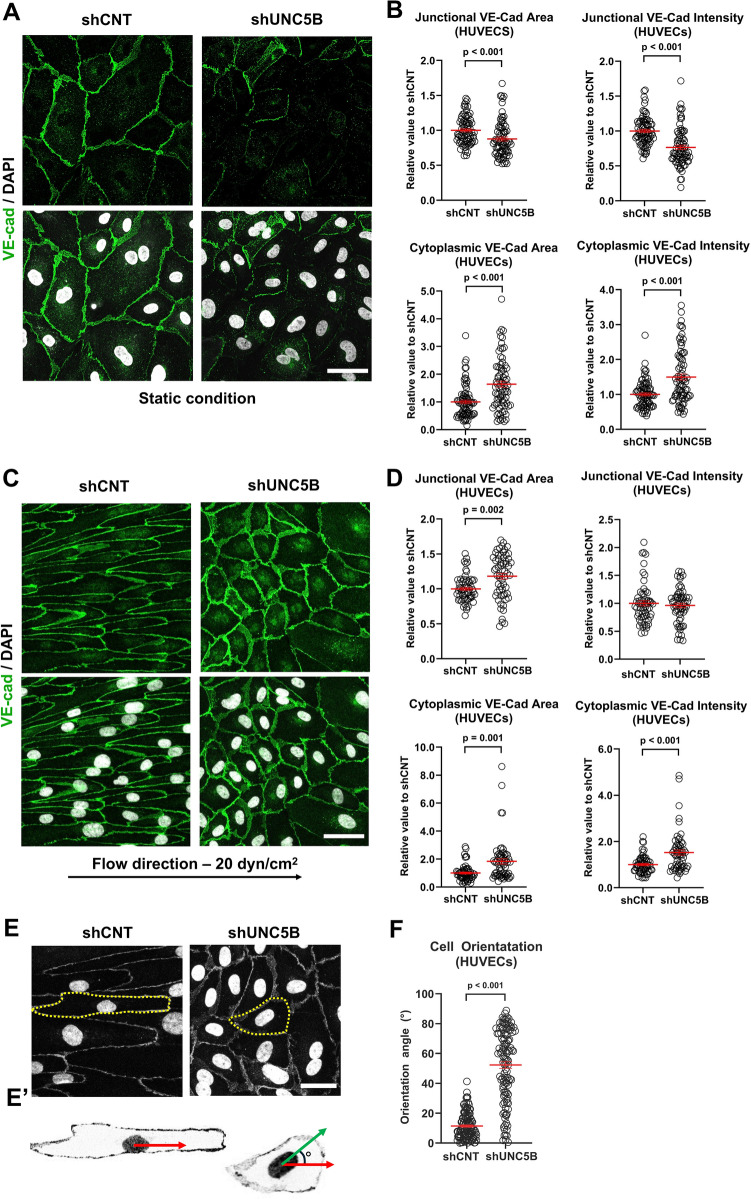
UNC5B regulates endothelial cell morphology, alignment, and junctional VE-cadherin under static and flow conditions. (**A**) Representative immunofluorescence images of shCNT and shUNC5B-treated HUVECs stained with anti-VE-cadherin (VE-cad, green) and DAPI (white). (**B**) Quantification of VE-cadherin area and fluorescent intensity at endothelial cell–cell junctions and endothelial cytoplasm under static conditions. (**C**) Representative images of shCNT and shUNC5B-treated HUVECs under high laminar flow (20 dyn/cm^2^). (**D**) Quantifying changes in VE-cadherin area at endothelial cell–cell junctions and endothelial cytoplasm under flow conditions. At least 60 representative fields of view were evaluated from three biological replicates for the graphs in B and D. (**E**) A representative schematic of the scoring for cell orientation angle. Direction of flow (red arrow) and cell axis (green arrow) are indicated. (**F**) Quantification of cell orientation angle in shCNT and shUNC5B cells under laminar flow. A total of 120 cells were evaluated per condition across three biological replicates. Unpaired t-tests, presented as mean ± s.e.m. Scale bars, 50 μm (**A**,**C**) and 33 μm (**E**).

Localization of VE-cadherin to the endothelial cell surface membrane is critical for endothelial cell–cell junction formation, which regulates migratory capacity. Notch signaling is required for the stabilization VE-cadherin junctions when evaluated in vitro and *in vivo*^27^. Examination of UNC5B protein expression revealed that UNC5B localizes throughout the cell, but is concentrated at endothelial membrane junctions, overlapping with VE-cadherin in HUVECs and mouse retinal vasculature (Supplemental Fig. 4D,E). To determine if UNC5B regulates VE-cadherin membrane association, we stained shCNT- and shUNC5B-transduced HUVECs for VE-cadherin and evaluated junctional and cytoplasmic staining under static monolayer culture. In HUVEC under static culture, *UNC5B* knockdown significantly lowered VE-cadherin area and intensity at endothelial junctions and significantly raised staining intensity in the cytoplasm (Fig. 4A,B). Total VE-cadherin levels did not change in shUNC5B-transduced HUVECs, indicating that *UNC5B* specifically regulates VE-cadherin membrane localization (Supplemental Fig. 4A,C).

To further characterize the role of UNC5B in VE-cadherin membrane localization, we examined VE-cadherin junctional stability using VE-cadherin tagged with photoconvertible protein Dendra2 at the C-terminus. We monitored VE-cadherin dynamics after photoconversion using a λ = 405 nm laser to induce an emission shift from λ = 488 to 543 nm (Supplemental Fig. 4F). Simultaneous imaging of the irradiated region enabled us to visualize the newly localized VE-cadherin-Dendra2 at λ = 488 nm and the photoconverted VE-cadherin-Dendra2 at λ = 594 nm over a 10 min timecourse (Supplemental Fig. 4G and Supplemental Video 1). We observed that shUNC5B-transduced HUVECs showed increased dissociation of VE-cadherin compared to shCNT-HUVECs (Supplemental Fig. 4H,I). This result demonstrates that UNC5B stabilized VE-cadherin assembly at adherens junctions.

Notch signaling is critical for endothelial response to shear stress. Arterial shear stress induces Notch signaling, which drives elongation of endothelial cell morphology, endothelial junction stabilization, and flow-directed cell orientation^29^. To determine if UNC5B is required for Notch-mediated junctional changes in response to shear stress, HUVECs transduced with shCNT- and shUNC5B were subjected to high laminar flow (20 dyn/cm^2^, Ibidi chip) for 72 h to mimic physiologic forces. Control HUVECs elongated in the direction of laminar flow (Fig. 4C). *UNC5B* knockdown cells showed a lack of cellular elongation and polarization, as evidenced by the absence of cellular alignment relative to the direction of flow (Fig. 4C,E,F). In response to flow, VE-cadherin forms a stable and organized network displaying less membrane overlap at cell junctions and overall linear distribution of VE-cadherin in control endothelial cells^58^. *UNC5B* knockdown HUVECs retained broad, overlapping endothelial junctions under laminar flow, as seen by a significant increase in area coverage by VE-cadherin staining (Fig. 4D). These data indicate that UNC5B is required for Notch-mediated stabilization of cell–cell junctions, maintenance of an elongated cellular morphology, and cell alignment with flow.

### Unc5B is regulated by Notch signaling in the mouse retinal endothelium

**Figure 5:**
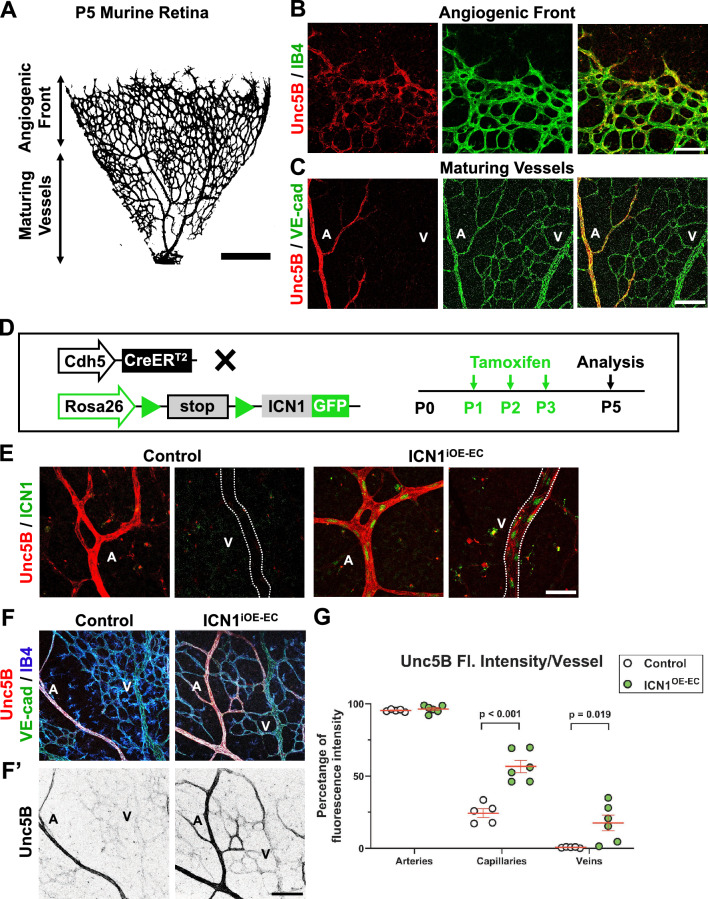
Endothelial cell-specific Notch activation increases Unc5B expression. (**A**) Representative postnatal day (P) 5 retina distinguishing the immature vascular plexus of the angiogenic front region from the central region, which has increasingly mature arteries, capillaries, and veins. (**B**) P5 retina stained with anti-Unc5B (red) and Isolectin B4 (IB4, green) to label the blood vessels at the angiogenic front. (**C**) P5 retina stained with anti-Unc5B and VE-cadherin (VE-cad, green) to label the maturing vessels. A = artery and V = vein in all panels. (**D**) Diagram of tamoxifen administration to ICN1^IOE-EC^ mice and harvest at P5 for analysis. (**E**) Detection of ICN1 in endothelial nuclei of ICN1^IOE-EC^ retina with anti-GFP antibody. (**F**) ICN1^IOE-EC^ mutant and control mouse retinas stained for Unc5B (red) and vasculature (VE-cad, green; IB4, blue). (**F’**) Isolated Unc5B channel depicted in grayscale. (**G**) Quantification of the percentage of Unc5B fluorescence intensity in arteries, capillaries and veins. At least 17 representative fields of view were examined and averaged from five distinct control mice and six distinct ICN1^OE-EC^ mice. Multiple comparisons unpaired t-test, presented as mean ± s.e.m. Scale bars, 230 μm (**A**), 100 μm (**B**–**E**), and 100 μm (**F**).

To evaluate if Unc5B is regulated by Notch in murine endothelium, we examined Unc5B expression in the developing mouse retina at a stage where there is active sprouting angiogenesis^59^. Angiogenic sprouts emerge at birth and progress outwards, permitting simultaneous and quantifiable visualization of the angiogenic front and maturing arteries, capillaries, and veins (Fig. 5A). In accordance with previous studies^45,49^, whole mount immunofluorescent (IF) staining with anti-Unc5B and vascular markers IsolectinB4 (IB4) or VE-cadherin showed that Unc5B is expressed in retinal arteries and branching capillaries, with scattered expression at the angiogenic front endothelium and little to no expression in the veins at P5 (Fig. 5B,C). These regions are consistent with known areas where Notch signaling is active^26^. To confirm that Unc5B can be regulated by Notch signaling in the retinal vasculature, we overexpressed ICN1 in retinal endothelium using the Cre-inducible ICN1-GFP allele *Gt(ROSA)26Sor*^*tm1 (Notch1)Dam*^*/J*^60^ in combination with the endothelial-specific tamoxifen-inducible Cre *Cdh5-CreER*^*T2*^. Tamoxifen was administered P1-P3 and retinas were isolated at P5 (Fig. 5D). To confirm Notch activation, whole-mount retinas from control mice (Cre^neg^; *Gt(ROSA)26Sor*^*tm1(Notch1)Dam*^*/J*^+^) and mice with ectopic endothelial ICN1-GFP expression (*Cdh5-CreER*^*T2*^*; Gt(ROSA)26Sor*^*tm1(Notch1)Dam*^*/J*^+^, abbreviated ICN1^iOE-EC^) were stained with anti-GFP antibody to confirm ICN1 expression (Fig. 5E). ICN1^iOE-EC^ retina showed reduced sprouting and vascular density of the retinal vasculature, as predicted from increased Notch signaling (Supplemental Fig. 5A)^26,31,54,61^. Activation of Notch signaling throughout the retinal vasculature by ICN1 did not significantly alter the staining intensity of Unc5B in arteries, but significantly increased ectopic Unc5B expression in veins and capillaries of ICN1^iOE-EC^ mice (Fig. 5F,G). We thus conclude that Unc5B expression can be regulated by Notch signaling in angiogenic vasculature.

### Endothelial-specific Unc5B knockout results in hypervascularization of retinal vasculature

**Figure 6:**
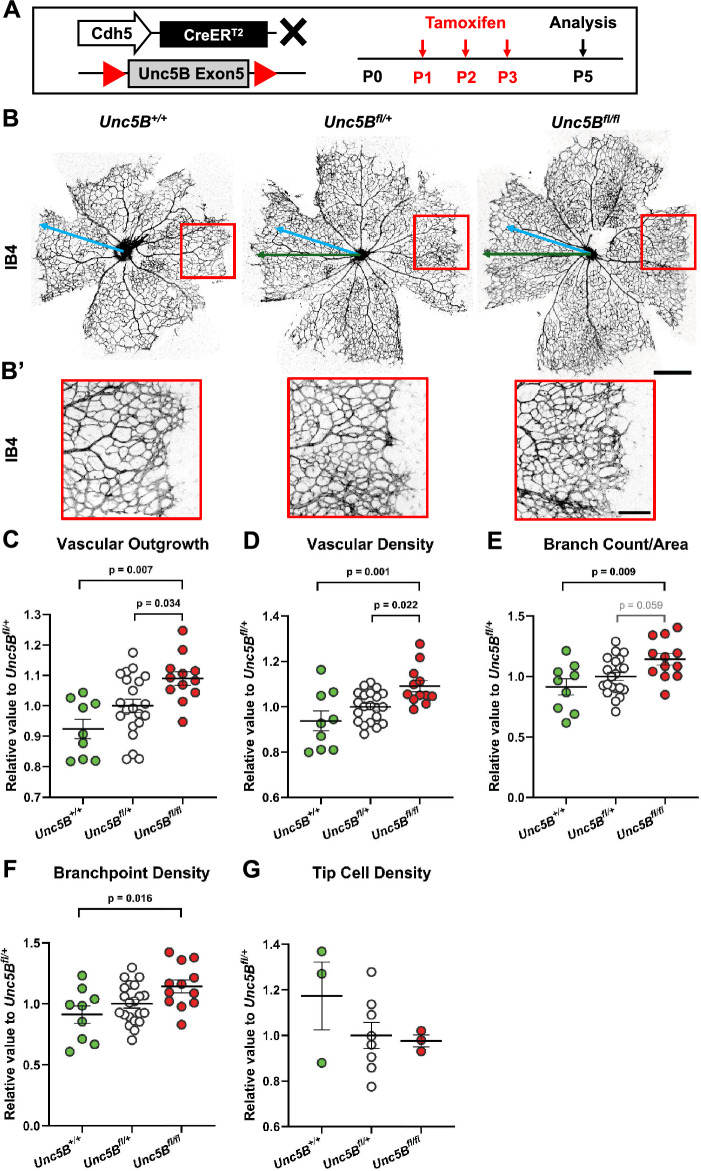
Endothelial Unc5B suppresses angiogenesis in the retina. (**A**) Diagram of tamoxifen administration to *Cdh5-CreER*^*T2*^*; Unc5B*^*flox*^ mice and harvest at postnatal day (P) 5 for analysis. (**B**) Whole-mount P5 retinas from *Unc5B*^+*/*+^, *Unc5B*^*fl/*+^, and *Unc5B*^*fl/fl*^ mice stained for the vasculature using Isolectin B4 (IB4, gray). Green arrows illustrate the degree of vascular outgrowth in *Unc5B*^*fl/*+^, and *Unc5B*^*fl/fl*^ retinas, while blue arrows represent the average vascular outgrowth of control retinas for comparison. Red boxes indicate magnified regions of the vasculature in B’. (**C**–**G**) Quantification of vascular outgrowth (**C**), vascular density (**D**), branch count per area (**E**), branchpoint density (**F**), and tip cell density (**G**) normalized to *Unc5B*^*flox/*+^. Each data point represents the average of 4–8 measurements on a single animal. One-way ANOVA, presented as mean ± s.e.m. from at minimum three distinct animals per genotype. Scale bars, 300 μm (**A**) and 100 μm (**A’**).

Loss of Notch1/Dll4 signaling in the neonatal retinal vasculature leads to hyperbranching in the capillary plexus and increased tip cells at the angiogenic front^26,62,63^. Global inhibition of Unc5B using neutralizing antibodies or null allele has shown similar phenotypes, but the endothelial-specific role of Unc5B has not been examined in detail in the retina^45–49^. To determine the role for Unc5B in endothelial cells during retinal angiogenesis, we combined a conditional allele of Unc5B^49^ (*Unc5B*^*flox*^) with *Cdh5-CreER*^*T2*^. Endothelial *Unc5B* excision was induced by administrating tamoxifen P1-P3 followed by retina harvest at P5 (Fig. 6A). Unc5B loss in endothelial cells was validated by IF staining of whole-mount retinas (Supplemental Fig. 5B,C). P5 retinas were stained with IB4 and an analysis of five key parameters of angiogenesis was conducted, including vascular outgrowth (distance the angiogenic front has expanded from the center), vascular density (percentage of field covered by IB4 staining), branch count (number of branch segments per unit area), branchpoint density (number of junctions per unit area), and tip cell density (number of tip cells per mm at angiogenic front) (Fig. 5B,B’). Cre-positive mice homozygous for Unc5B^flox^ (Unc5B^fl/fl^ mutant mice) were compared with Cre-positive Unc5B^flox^ heterozygous (Unc5B^fl/+^) and Unc5B wildtype (Unc5B^+/+^) littermate controls, with each litter normalized to average heterozygous value to control for litter growth differences. We observed significant increases in vascular outgrowth, density, branch count, and branchpoint density in Unc5B^fl/fl^ mutant mice compared to controls (Fig. 6C–F). Unc5B heterozygous animals showed an intermediate phenotype which trended toward increased vascularization but did not differ significantly from controls. No significant observations were observed when tip cell density was quantified (Fig. 6G). These results indicate that Unc5B is a negative regulator of angiogenic outgrowth and branching, but not angiogenic tip sprouting, consistent with a role for Unc5B as an effector of specific Notch phenotypes.

## Discussion

Notch signaling is essential for a wide range of biological processes, including angiogenesis^26,64^. Notch mediates angiogenic growth by limiting endothelial proliferation and migration and enhancing endothelial cell–cell adhesion to promote stalk cell attributes and vessel maturation^26,27,54,55,62,63^. Notch proteins function as transcription factors, but the downstream effectors that convert Notch transcriptional activity into specific endothelial behaviors are incompletely understood. In this study, we identified novel endothelial genes that respond to the gain or loss of Notch signaling during angiogenesis and revealed UNC5B as a novel Notch effector.

We established that *UNC5B* was regulated by Notch within a few hours of Notch activation or inhibition in multiple cellular contexts. *UNC5B* is upregulated by Notch activation via ligand presentation or the chelating agent EGTA and downregulated by γ-secretase inhibition. Knockdown of *UNC5B* shows many similar phenotypes as inhibition of Notch signaling in cultured endothelial cells, including de-suppression of endothelial proliferation and migration. We confirmed that *UNC5B* acts downstream of Notch signaling in these phenotypes by ectopically activating Notch signaling and confirming that loss of *UNC5B* is sufficient to block endothelial response to Notch signaling.

One of the major physiologic roles of Notch signaling is maintaining arterial endothelial phenotype. Arterial hemodynamic forces, in particular laminar shear stress, induce endothelial elongation, reassembly of adhesion junctional complexes, and cytoskeletal reorganization^29,58,65^. High laminar shear stress is associated with an “atheroprotective” endothelial gene profile that is protective against the development of atherosclerosis^66^. Notch signaling is induced by laminar shear force and is required for the maintenance of junctional integrity and endothelial cell elongation in response to flow^29^. We have established that *UNC5B* is required to stabilize endothelial cell–cell junctions under both static and flow conditions and is required for endothelial elongation in response to flow. We observed that the absence of *UNC5B* in laminar flow conditions increased VE-cadherin junctional area, indicating increased membrane protrusive behavior and reduced junction stability. We also observed failure in endothelial cell orientation in response to laminar flow. These findings indicate that *UNC5B* plays a critical role in these Notch-mediated phenotypes and suggest that further research is warranted into the potential role of *UNC5B* in atheroprotection.

In addition to the arterial vasculature, Notch signaling and Unc5B expression are found in vascular sprouts during angiogenesis^26^. Genetic and pharmacological inactivation of Notch signaling leads to hypervascularization, as previously demonstrated by the formation of highly branched and dense vascular networks^26,62,63^. Endothelial cell-specific deletion of *Unc5B* also led to a hypervascularization phenotype, as indicated by the increase in vascular outgrowth, vessel density, branchpoint density, and the number of branchpoints. A recent preprint from the Boyé lab also examines the role of *Unc5B* in the developing retinal vasculature, but conversely observes a decrease in vascular outgrowth and density^67^. This discrepancy may be due to earlier induction of *Unc5B* excision. Notch-induced endothelial outcomes are biphasic – loss of Notch signaling causes a short-term increase in sprouting angiogenesis, but causes cell cycle arrest and angiogenic failure over longer developmental timeframes^31^. Both observations are consistent with the hypothesis that Notch signaling regulates vascular development by inducing UNC5B to control angiogenic growth.

Notch signaling upregulates a large number of transcriptional targets, which suggests that different targets act as effectors for different Notch-mediated endothelial phenotypes, and/or multiple effectors may play overlapping roles. Inhibition of Notch signaling in the developing retinal vasculature triggers excessive capillary branching and formation of tip cells^26^. We observed excessive capillary branching, but no differences in tip cell count, in Unc5B^fl/fl^ mice. This suggests that Unc5B specifically mediates capillary branching but does not mediate Notch regulation of tip/stalk cell identity or may act redundantly with another Notch effector.

Some previous studies have shown that Notch4 has an overlapping and redundant role with that of Notch1 in some vascular beds^64,68^, but other studies have suggested that Notch4 is a weaker transcriptional activator than Notch1 and may even act as an inhibitor of Notch1 function in some cell types^69^. In primary human endothelial cells, we observed comparable upregulation of *UNC5B* by activated Notch1 and Notch4 proteins. However, we found that *UNC5B* is required for Notch1-mediated, but not Notch4-mediated, suppression of endothelial migration and is required for Notch4-mediated, but not Notch1-mediated, suppression of endothelial proliferation. These results suggest that Notch1 and Notch4 may regulate these phenotypes to various degrees and may use different downstream effectors for some functions.

The mechanism(s) of UNC5B regulation of endothelial phenotypes are still incompletely understood, but *UNC5B* promotion of VE-cadherin stabilization at adherence junctions may play a key role. Junctional stabilization is required for endothelial shear stress response, which was blocked in *UNC5B* knockdown cells^58^. Conversely, destabilization of VE-cadherin at adherens junctions promotes endothelial proliferation, migration, and angiogenic sprouting and vessel branch formation *in vivo*^70^, all of which were upregulated in *UNC5B* knockdown cells and *Unc5B* knockout mice. It is therefore likely that UNC5B contributes to Notch phenotypes largely via regulation of VE-cadherin in stabilizing the vascular junctions and preventing aberrant angiogenesis.

Notch signaling regulates a large number of endothelial genes, many of which may play unique, overlapping, or redundant roles as effectors^52^. Another Notch effector recently identified by our lab, *RND1*, was significantly downregulated in GSI-treated RiboTag brain samples, although it was not as strongly regulated as *UNC5B* (-0.3 Log2 fold change, padj = 0.02)^52^. Like *UNC5B*, *RND1* is required for Notch-mediated suppression of endothelial migration and sprouting, but loss of *RND1* had no effect on endothelial proliferation. *RND1* is a member of the Rho GTPase family, and was found to facilitate Notch suppression of Ras activity, which is crucial for mediating cell adhesion and actin cytoskeleton organization. The observation that both of these genes regulate migration and sprouting, one via membrane junctions and one via cytoskeletal rearrangements, highlights the importance of specific cell biologic alterations in enacting the phenotypic changes associated with Notch signaling. Identification of new Notch effectors and better understanding of the contexts in which specific effectors are induced will be instrumental in understanding the various mechanisms by which Notch regulates endothelial function and sprouting angiogenesis.

Angiogenesis is a developmental process that requires tight coordination of endothelial cells to form new blood vessels. Pathological angiogenesis is a hallmark of many diseases, including diabetic retinopathy, cardiovascular diseases, and cancer^1–5^. Notch signaling directly regulates a cohort of genetic effectors to control angiogenic growth such as UNC5B. Our study highlights UNC5B as a downstream effector of Notch signaling which regulates endothelial proliferation, junctional stability, and capillary branching. Approaches to enhance UNC5B signaling could promote vascular stabilization and meet the therapeutic needs of angiogenesis-dependent diseases.

## Methods

### Methods reporting

All the experimental methods in this study were performed in accordance with the relevant guidelines and regulations and reported in accordance with ARRIVE guidelines.

### Mice

All mouse experiments were performed according to the Animal Care Committee guidelines at the University of Illinois Chicago under approved protocols and comply with the USPHS Policy on humane care according to the *Guide for Care and Use of Laboratory Animals*^71^. All mice were maintained on a C57BL/6 J background. Female and male mice were used interchangeably in these studies.

*Cdh5(PAC)-CreER*^*T*2^ mice were bred into a background of homozygous *RiboTag*^*floxstop*^ (*Rpl22*^*tm1.1Psam*^, JAX: 029,977) mice^50^ to create mice with EC-specific ribosome labeling (RiboTag^EC^). *Cdh5-CreER*^*T2*^ mice were also bred with *Unc5B*^*flox*^ (*Unc5B*^*tm1(flox)Slac/Slac*^) mice^49^ and *ICN1*^*floxstop*^ (*Gt(ROSA)26So*^*rtm1(Notch1) Dam/J*^*,* Jackson Labs strain #008159) mice^60^ to generate mice with endothelial cell (EC)-specific loss of *Unc5B* and EC-specific active Notch signaling. The *RiboTag*^*floxstop*^ mice were provided by Peter Canoll (Columbia University Pathology Department, New York, New York, USA) and *Cdh5(PAC)-CreER*^*T2*^ mice were obtained from Ralf Adams (Max-Planck Institute for Molecular Biomedicine, Germany).

To induce Cre-mediated gene recombination, tamoxifen (TMX; Sigma, T5648) was dissolved in corn oil (Sigma, C8267), and then 100 µg/pup/day was injected peritoneally at postnatal day (P) 1-P3 for early induction. TMX-injected *Cdh5-CreER*^*T2*^*;Unc5B*^*wt/wt*^ and *Cdh5-CreER*^*T2*^*;1ICN1*^*wt/wt*^ littermates were always used as controls. Mice were humanely euthanized via decapitation with a sharp blade.

All genotyping was performed by Transnetyx.

### Mouse brain isolation and immunofluorescence

Brains were isolated at P8 from RiboTag^EC^ mice and fixed overnight in 4% Paraformaldehyde (Thermo Fisher Scientific) overnight at 4 °C. Following fixation, samples were cryoprotected using 30% sucrose solution in PBS for 24 h at 4 °C. Samples were embedded into OCT (Tissue Tek) and sectioned at 10 μm using a cryostat (Thermo Scientific, Cryostar Nx50). Cryosections were warmed at room temperature and then blocked in 3% BSA and 2% donkey serum. Following permeabilization, primary antibodies listed in Supplemental Table 2 were diluted in the blocking solution overnight at 4 °C followed by incubation with species-specific Alexa Flour-coupled secondary antibodies (Supplemental Table 2) for 30 min at room temperature. After incubation with secondary antibodies, cryosections were mounted in Vectashield mounting medium with DAPI (Vector Laboratories).

### Isolation of polysome-bound mouse brain endothelial mRNA using RiboTag

RiboTag^EC^ were treated intragastrically with 100 µg/pup/day TMX from P1-P3 to induce endothelial cell-specific ribosome labeling. At P8, mice were injected subcutaneously with a single dose of 100 mg/kg of GSI DAPT (20 mg/ml in 10% ethanol and 90% corn oil) or vehicle control (CNT) alone. 6 h following injection, pups were humanely euthanized. Forebrains were isolated and immediately snap frozen in 1.5 ml microcentrifuge tubes in liquid nitrogen and stored at -80 °C for later use.

Frozen brains were weighed to create a 5% weight/volume homogenate ratio to calculate the amount of Homogenizing Buffer required per sample. The Homogenizing Buffer consists of 10 mM Tris pH7.5, 50 mM NaCl, 15 mM MgCl_2_, 1 mM DTT, 0.5% Triton-X-100, 100 μg/mL Cyclohexamide. For every 1 ml, we supplemented the homogenizing buffer with 12 μl Superase Inhibitor (Invitrogen, AM2696), 12 μl turbo DNase (Invitrogen, AM2238), and 10 μl Protease Inhibitor (Thermo Scientific, 78430). Samples were homogenized using G27 needles. After the tissue was completely homogenized, Postmitochondrial supernatant was formed by centrifugation at 10,000 rpm for 10 min at 4 °C. 50 μl of the supernatant was saved for the input.

For immunoprecipitation (IP) against HA, we added 4 μl of anti-HA-tag antibody (Abcam, Supplemental Table 2) to 800 μl of each sample prior to incubation on an orbital shaker for 4 h at 4 °C. An hour before the end of incubation, 350 μl of Dyna Protein A magnetic beads (Invitrogen, 10001D) were collected from the stock solution and were equilibrated in 800 μl of the homogenizing buffer for 1 h at 4 °C. Following completion of incubation, the brain supernatant containing the anti-HA antibody was transferred to the equilibrated magnetic beads for incubation overnight on an orbital shaker at 4 °C. The next day, IP beads were washed three times with a wash buffer (1 ml homogenizing buffer, 6 μl Superase Inhibitor, 6 μl turbo DNase, and 5 μl Protease Inhibitor) and resuspended in 350 μl of RLT plus β-Mercaptoethanol. Total RNAs were extracted using a RNeasy mini kit (Qiagen) according to manufacturer’s instructions.

### RNA-sequencing and gene expression analysis of RiboTag samples

RNA quantity and integrity were measured using Bioanalyzer (Agilent TapeStation 4200, UIC Genome Research Core) prior to RNA sequencing. The RNA isolated from the forebrain were sequenced were sequenced at a depth of ~ 30 million 100-base single-end reads on the TruSeq platform (Sulzberger Columbia Genome Center).

Reads from IP-ribosomes were aligned with the mouse rRNA reads with STAR (version 2.5.2b) to remove the contamination of the ribosomal RNA reads from the mRNA reads. Next, the reads were mapped to the mouse transcriptome Mouse (UCSC/mm10) using STAR and processed with Samtools to generate the bam files. The bam files were processed to obtain raw counts by FeatureCounts (version 1.5.0-p3), to generate a table of counts for each gene in the genome.

These raw counts were normalized and then tested for differential gene expression to identify Notch targets in the endothelium. To do so, we used DESeq2 (version 1.18.1)^72^, a R Bioconductor package which utilizes a generalized linear model that can infer the effects of individual factors and their interactions in experiments with multifactorial design. The normalized counts were scaled to a log scale and were used to generate the principal component analysis (PCA) and the Volcano plots using DESeq2. The PCA segregated the samples into four clusters based on their library type: (1) homogenate CNT, (2) IP CNT, (3) homogenate GSI, and (4) IP GSI (Supplemental Fig. 1A).

The RNAseq datasets generated during this study are available in the NCBI Gene Expression Omnibus repository at https://www.ncbi.nlm.nih.gov/geo/ (Accession number GSE163568).

### Cells

All cell cultures were maintained at 37 °C in a mixture of 5% CO_2_ and 95% humidified air.

Primary Human Umbilical Vein Endothelial Cells (HUVECs) were purchased from Promocell and grown on EGM2 Media on culture plates coated with rat tail type I collagen (BD Biosciences, 354236). HUVECs used for experiments were of passage six or lower.

Primary Human Retinal Microvascular Endothelial Cells (HREC; Cat. ACBRI 181, Passage 3) were obtained from a single vial purchase from Cell Systems. HRECs were cultured in EGM-2 MV Media (Lonza) with all bullet kit components on dishes coated with 0.2% gelatin. Media was changed every other day until the cells reached 80% confluency. HRECs used for experiments were of passage 8 or lower.

Primary Pulmonary Microvascular Endothelial Cells (PMVECs, Cat. No. C-12281, Passage 2) were obtained from a single vial purchased from Promocell. PMVECs were cultured with Endothelial cell growth medium (EGM) MV (Promocell) with all components of the supplemental bullet kit on dishes coated with 0.2% gelatin (Sigma, G1393) at passage six or lower. Media was changed every other day until the cells reached 80% confluency.

HEK293T cells were purchased from ATCC and maintained in DMEM (Gibco, 11–995-073) with 10% FBS.

### Tethered ligand assay (TLA)

The TLA was performed as previously described^52^. 10 μg/ml of the recombinant extracellular domains of the Notch ligands hDLL4-Fc (Sino Biologicals Inc, 10,171-H202H) or IgG-Fc (Sino Biologicals Inc, 10,702-HNAH) were coated on 24-well plates (Corning, 353226) in a Fibronectin matrix (10 μg/ml, Sigma, F1141). Primary ECs were incubated overnight at 4 °C, trypsinized at 80% confluency, and seeded onto the coated plates. 6 h after incubation, RNA was isolated.

To inhibit endogenous Notch signaling, we treated primary ECs at 70% confluency with a GSI inhibitor, Compound E (CpE; Enzo Life Sciences, ALX-270–415-c250) at 500 nM. CpE was added to media at the same time as cells were seeded.

### EGTA Notch activation assay

The EGTA Notch activation assay was performed as previously described^52^. Briefly, primary ECs at 70% confluency were treated with CpE at 200 nM overnight. The next day, ECs were treated with DPBS containing 10 mM EGTA for 15 min at 37 °C after which the DPBS was replaced with fresh EGM-2 (media for HUVECs) or EGM-2 MV (media for HRECs). RNA was collected at 1, 1.5, 2, 4, and 6 h after adding EGTA. For the CpE treatment group, 500 nM of CpE was added with EGTA at 0 h.

### Generation and validation of ICN overexpression in primary ECs

To perform stable overexpression and knockdown studies in primary ECs, a third-generation lentiviral infection system was used. To activate Notch signaling, we created a human Notch1 and Notch4 intracellular domain (ICN1 and ICN4) overexpression lentivirus vectors (pCCL-PGK-ICN1 and pCCL-PGK-ICN4) consisting of human ICN1 and ICN4 in a pCCL vector with a PGK promoter. tdTomato (RFP) expressing vectors were used as a control. Lentivirus was generated in HEK293T cells through co-transfection of lentiviral packaging (5 μg pMDLg/pRRE and 2.5 μg pRSV-Rev) and envelop vector (3 μg of pCMV-VSV-g) with 10 μg of pCCL-PGK-RFP, pCCL-PGK-ICN1, or pCCL-PGK-ICN4. For lentivirus transduction, HUVECs were incubated with EGM-2 and the supernatant of the lentivirus transfected HEK293T cells (2:1 ratio mixture) overnight and changed to fresh EGM-2 media the next day. To confirm the expression levels of the ICN variants and canonical Notch targets, RNA was isolated from the HUVECs 24 h after transduction of lentivirus using the RNeasy Mini Kit (Qiagen, 74104) according to the manufacturer’s instructions and qRT-PCR was performed with the primers listed in Supplemental Table 1.

### Knockdown of Unc5B using shRNA

To knockdown *Unc5B*, lentivirus containing pLKO.1-U6-shUNC5B (Sigma), targeting the coding sequence of Unc5B, or empty pLKO.1-U6-scramble B (shCNT; Addgene, #1864) vector. Lentivirus of shCNT and shUNC5B were packaged and used to infect primary ECs as described above. shCNT expressing vectors were used as a control. *Unc5B* knockdown was validated by RT-qPCR using primers listed in Supplemental Table 1. The sequences for shUNC5B and shCNT plasmids are provided below:

shUNC5B: CCGGCAGAAGATATGCAACAGCCTACTCGAGTAGGCTGTTGCATA

TCTTCTGTTTTTG

shCNT: CCGGTCCTAAGGTTAAGTCGCCCTCGCTCGAGCGAGGGCGACTTA

ACCTTAGGTTTTTG

### Quantitative real-time polymerase chain reaction (qRT-PCR)

RNA isolated from primary ECs was collected using Qiagen RNEASY according to the manufacturer’s recommendations. cDNA was generated using approximately 1 μg RNA per 20 μl using the Verso cDNA Synthesis Kit (Thermo Fisher Scientific, AB-1453). Quantitative PCR (qPCR) was performed on ABI ViiA7 real-time PCR system (Life Technologies) using the Fast SYBR Green Master Mix (Applied Biosystems). The primers used are listed in Supplemental Table 1. As indicated in the figure legends, normalized transcript levels are relative to the levels of β-actin. All relative gene expression analyses were performed using the comparative C_t_ method with triplicate reactions for each sample evaluated.

### Western blots

Primary endothelial cells were lysed in ice-cold RIPA buffer (Cell signaling, 9806) containing 1 × protease inhibitor (Thermo Fisher Scientific, 78430), 1 × phosphatase inhibitor (Thermo Fisher Scientific, 78420), and 1 mM of DDT. Western blots were performed by loading 20–40 μg of protein onto 4–20% precast gels (Bio-Rad). Primary antibodies against UNC5B, VE-CADHERIN, and α-TUBULIN (Supplemental Table 2) were incubated in a blocking buffer (5% BSA, 1 × TBST, and 0.1% Tween 20) and HR-conjugated secondary antibody (Cell Signaling Technology, 70745) was used for detection with ECL (GE Amersham, RPN2209). Gel images were obtained using the Chemidoc MP Imaging System (Bio-Rad) and quantification was performed using ImageJ.

### Scratch wound-migration assay

HUVECs were transduced with lenti-shCNT and shUNC5B and seeded in 24-well plates. A “scratch-wound” was created by removing a line of cells using a 200 μl pipette tip. After wounding, microscopy was used to image cell migration to the scratch closure for 22 h. To generate a percent closure score, the remaining open area was divided by the original open area and normalized to the control group (shCNT).

PMVECs were transduced with lentivirus of shCNT, shUNC5B, RFP, ICN1, and ICN4 and seeded in 24-well plates. Percent closure was measured at a single timepoint 6–9 h after wounding and normalized to the control group (shCNT-RFP).

### MTT viability assay

To measure primary EC proliferation, 96-well plates were seeded with 4 × 10^3^ HUVECs or PMVECs. After incubation for 72 h, cell number was determined using the MTT assay (Sigma). To analyze cell viability, the absorbance value was measured with 450 nm wavelength, which was subtracted from the 650 nm wavelength (Background signal) in the Microplate reader (Spectramax). The relative EC number was normalized by the control group (shCNT-RFP).

### Immunofluorescent staining of HUVECs

HUVECs were fixed in 4% Paraformaldehyde for 10 min at room temperature. After fixation, cells were permeabilized and blocked in 3% BSA (Fisher Bioreagents) and 0.02% Triton-X-100 (Fisher Bioreagents) in PBS with the primary antibodies overnight listed in Supplemental Table 2. Following staining with primary antibodies listed in Supplemental Table 2, Cells were incubated for 1 h at room temperature with species-specific Alexa Flour-coupled secondary antibodies (Supplemental Table 2) in blocking solution. After incubation with secondary antibodies, Vectashield Plus Antifade Mounting Medium with DAPI (Vector Laboratories) was added to the cells.

### Laminar flow assay

Laminar shear stress was applied to HUVECs as previously described^29^. Three independent experiments were conducted by seeding monolayers of HUVECs transfected with either shCNT or shUNC5B in µ-Slide I 0.4 Luer ibiTreat chambers (ibidi #80176). Cells were subjected to unidirectional constant laminar flow at 20 dyn/cm^2^ using the ibidi pump system (ibidi #10902) for 72 h in a 37 °C incubator with 5% CO_2_. After 72 h, HUVECs were immediately fixed with 4% Paraformaldehyde and prepared for immunofluorescence staining using the method described above. Static monolayers of HUVECs were cultured alongside flow-treated monolayers.

### VE-cadherin-Dendra2 photoconversion assay

To study VE-cadherin dynamics, shCNT and shUNC5B-treated HUVECs expressing the photoconvertible protein VE-cadherin-Dendra2 were imaged at 5% CO_2_ at 37 °C with λ = 488 nm and λ = 543 for the green and red states of Dendra2, respectively, after irradiation using a λ = 405 nm laser at 8–12% power as previously described^73^.

### Mouse retina isolation and immunofluorescence

All immunostainings of mouse retina were performed as previously described^74^. Eyes were enucleated following mouse sacrifice at P5. Collected eyes were fixed in 4% Paraformaldehyde for 1 h at 4 °C. Following fixation, eyes were washed with ice-cold 1X PBS solutions. Retinas were dissected and permeabilized in 1% BSA (Fisher Bioreagents) and 0.5% Triton-X-100 (Fisher Bioreagents) in PBS at 4 °C. Primary antibodies were diluted in blocking solution (5% Triton-X-100, 1 M MgCl_2_, 1 M CaCl_2_, and 1 M MnCl_2_ in PBS) and incubated with retina overnight at 4 °C. Following staining with primary antibodies listed in Supplemental Table 2, retinas were incubated for 2 h at room temperature with species-specific Alexa Flour-coupled secondary antibodies (Supplemental Table 2) in blocking solution. After incubation with secondary antibodies, immunostained retinas were postfixed with 4% Paraformaldehyde and flat-mounted in Vectashield mounting medium (Vector Laboratories).

### Image acquisition and data analysis

Brain fluorescent images, whole-mount fluorescent retinal images, and HUVEC brightfield images were acquired using the Leica DMi8 fluorescent microscope. Confocal stacked fluorescent retinal images and HUVEC images were captured using the Zeiss LSM 880 Confocal Microscope with Airyscan. All images were analyzed using the Image J software (NIH).

For VE-cadherin area and intensity quantification under static and laminar flow conditions, fluorescent images were thresholded using the IsoData algorithm on ImageJ. Selections were created for the threshold pixel, which represented junctional VE-cadherin and the mean gray value, area and integrated densities were measured for each individual image. For cytoplasmic VE-cadherin, the selections were inverted and the same measurements as junctional VE-cadherin were performed for each image.

For HUVEC orientation measurement under flow, the angle tool in ImageJ was utilized to quantify the alignment of endothelial cells in the direction of flow. Using this tool, a line was drawn parallel to the direction of flow for each endothelial cell. Then, without releasing the mouse button, another line was drawn parallel to the long-cell axis, as illustrated in Fig. 6E’.

To calculate VE-cadherin dynamics at adherens junctions using VE-cadherin-Dendra2, images in green and red channels were simultaneously acquired every 15 s using a Zeiss LSM 880 confocal microscope. The dissociation rate constants were calculated from recovery kinetics using nonlinear regression to fit the values to the one-phase association equation Y = Y_0_ + (Plateau – Y_0_) x [1-exp(-k * t)], where Y = 543 fluorescent intensity, Y_0_ = initial fluorescent intensity, Plateau is the minimum fluorescent intensity after photoconversion, and t = time.

For vascular outgrowth, measurements were taken from the center of the retina to the tip of the peripheral vessels and averaged per mouse. For vascular density, branch count, branchpoint density, and tip cell density, fields of view (1042.9 μm × 260.7 μm) focused on the angiogenic front plexus were used for quantification. At least 8 fields were analyzed per retina. Fluorescent images were thresholded using the ImageJ IsoData Dark algorithm on ImageJ. Vascular density was quantified by measuring the area of vessels in each image selection. Branchpoint density was quantified using the same selection as vascular density but was performed by measuring the number of junctions in each field with the ImageJ skeletonization tool. Prior to the junction measurement, images were despeckleled and smoothened, and the contrast was enhanced. A bandpass filter was then applied to filter out cells and other matter in the background. Huang dark was used for auto-thresholding. Lastly, skeletonization with pruning of the lowest intensity branches was completed. Tip cells were counted manually, and tip cell density was determined by dividing the number of tip cells by the total length of the angiogenic front. To quantify Unc5B in the retina, fluorescent intensity was thresholded and measured per field area.

### Statistical analysis

All data were plotted and analyzed in GraphPad Prism 9. Unless otherwise noted, all quantified data were either analyzed using unpaired *t*-tests or a one-way ANOVA. The number of animals used per experiment for in vivo studies and the number of experimental replicates performed for in vitro studies is provided in the figure legends. Significant differences have been reported in the figures and error bars represent the standard error of the mean. All data has been normalized to the experimental control identified in each figure.

### Supplementary Information

Supplementary Information.

Supplementary Video 1.

## Data Availability

RNA sequencing data is available in the NCBI Gene Expression Omnibus repository at https://www.ncbi.nlm.nih.gov/geo/ (Accession number GSE163568). Other data and materials that support the findings of this study are available from the corresponding author (L.A.N.) upon reasonable request.
