# Supplementary material for: Notch signaling regulates UNC5B to suppress endothelial proliferation, migration, junction activity, and retinal plexus branching
Source: Sci Rep. 2024 Jun 13;14:13603. doi: 10.1038/s41598-024-64375-z (PMC11169293; doi:10.1038/s41598-024-64375-z)
Supplement: Supplementary file 1 — Supplementary Information. [file 41598_2024_64375_MOESM1_ESM.docx]

**TITLE:**

**Notch signaling regulates UNC5B to suppress endothelial proliferation, migration, junction activity, and retinal plexus branching.**

**AUTHORS:**

Qanber Raza^1^, Taliha Nadeem^1^, Seock-Won Youn^1^, Bhairavi Swaminathan^1^, Ahana Gupta^1^, Timothy Sargis^1^, Jing Du^1^, Henar Cuervo^2^, Anne Eichmann^3^, Susan L. Ackerman^4^, L.A. Naiche^1*^, Jan Kitajewski^1, 5^

^1^ Department of Physiology and Biophysics, College of Medicine, University of Illinois at Chicago

^2^ Centro Nacional de Investigaciones Cardiovasculares Carlos III- CNIC- (F.S.P), Madrid, Spain.

^3^ Yale School of Medicine, New Haven, Connecticut

^4^ University of San Diego, San Diego, California

^5^ University of Illinois Cancer Center

***Corresponding Author:**

L. A. Naiche

University of Illinois at Chicago

Department of Physiology and Biophysics

1853 W Polk St, Rm 522 (MC 901)

Chicago, IL 60612

Phone: (312) 996 – 7620

Email: [naiche@uic.edu](mailto:kitaj@uic.edu)

**Supplemental Figure 1**

**
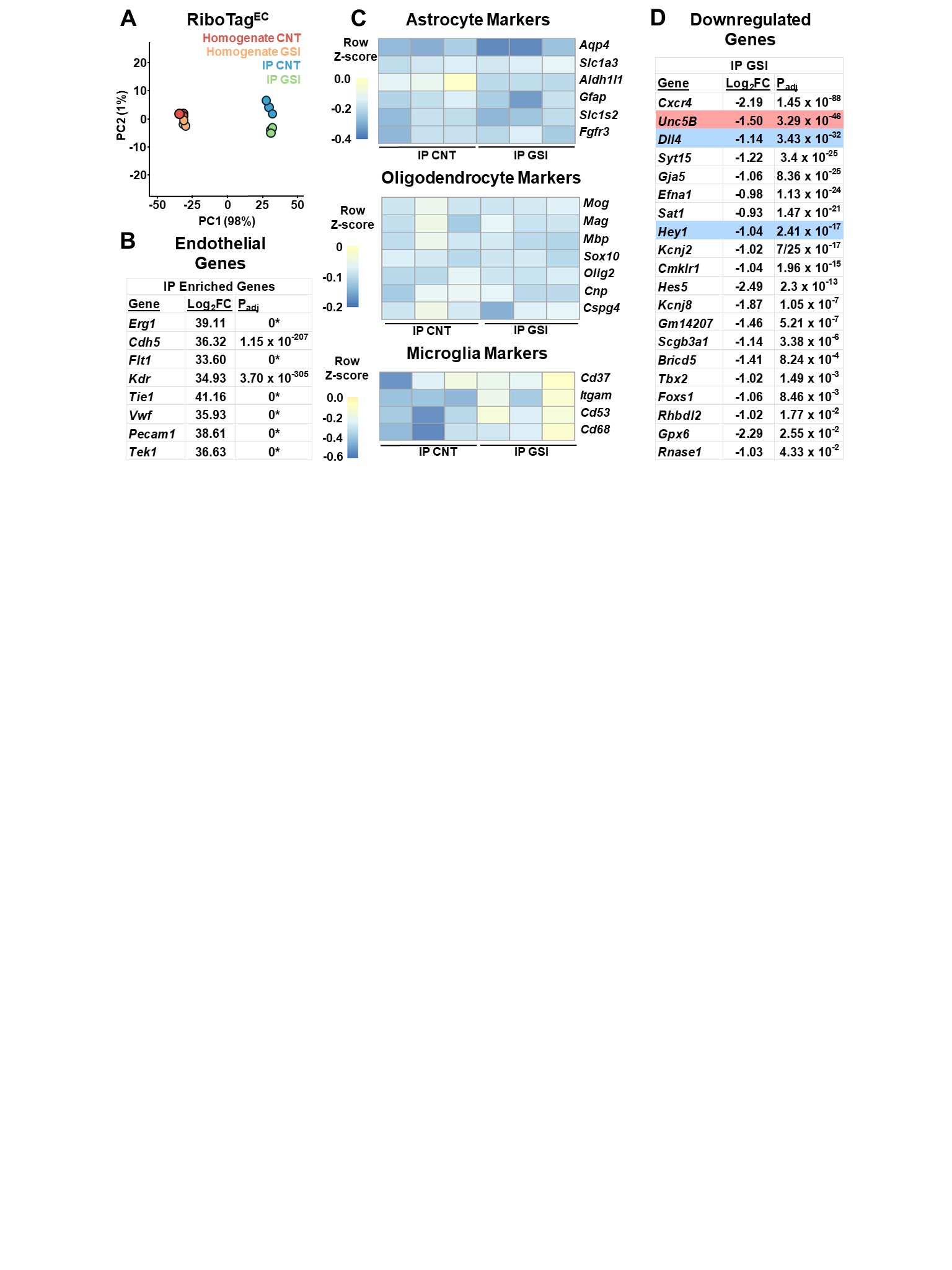
**

**Supplemental Figure 1. Immunoprecipitation of tagged ribosomes strongly enriches for endothelial transcripts.** (A) Principal component analysis (PCA) plot illustrating gene expression profile from homogenate and IP fraction from control (CNT) and GSI-treated RiboTag^EC^ mice. (B) Fold enrichment for endothelial transcripts of RiboTag IP fraction compared to bulk brain homogenate. (C) Depletion of non-endothelial brain cell types in RiboTag IP fraction. (D) Fold enrichment for the 20 most significantly downregulated transcripts of RiboTag IP GSI treated fraction compared to CNT treated fraction. Known Notch targets *Hey1* and *Dll4* (blue) and novel target *Unc5B* (red) were significantly downregulated in GSI-treated animals. All heatmaps represent z-score, as indicated. * = p values below computational threshold of the DESeq2 software.

**Supplemental Figure 2**

**
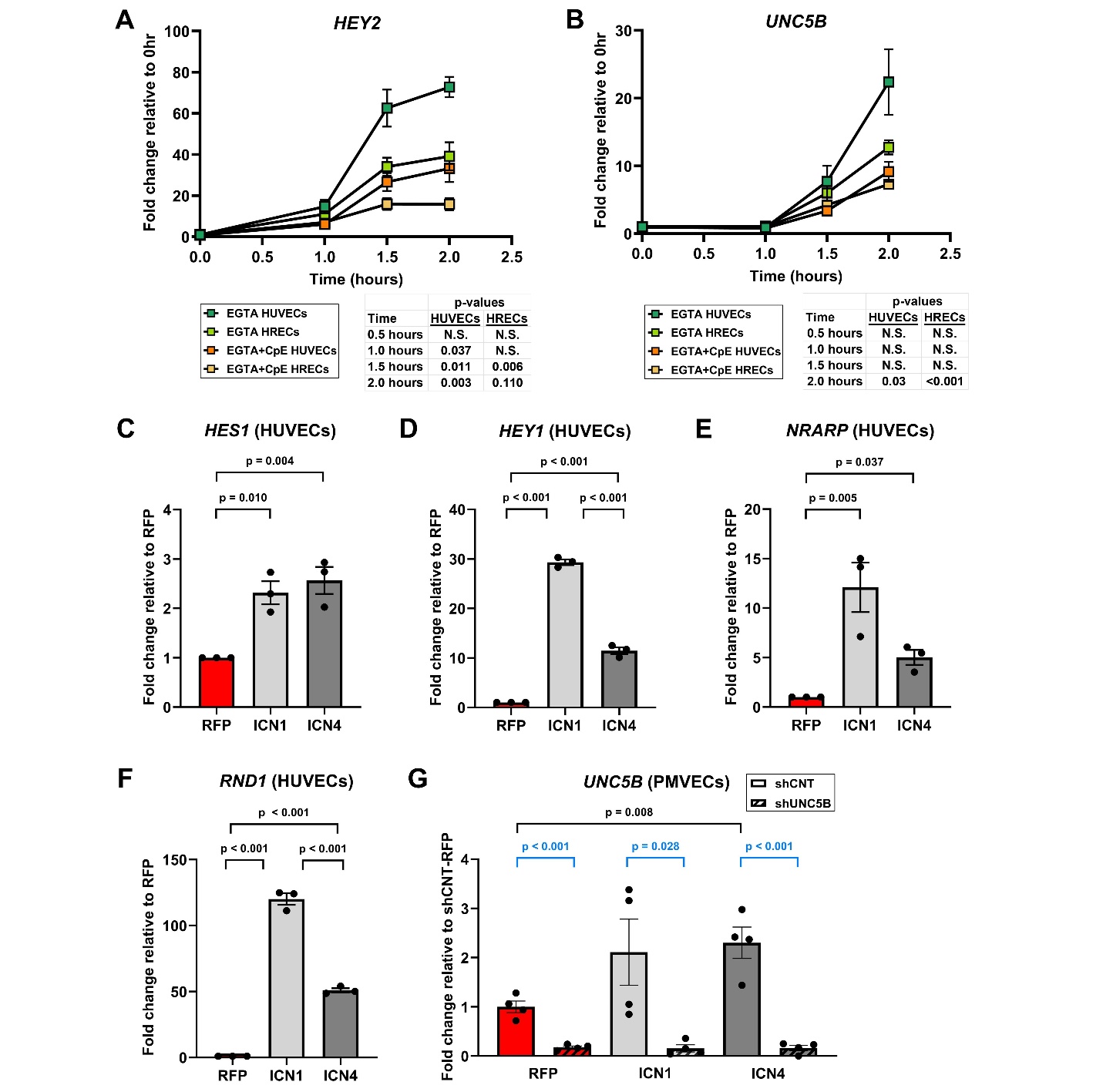
**

**Supplemental Figure 2. Notch activation using EGTA or Notch intracellular domain expression upregulates expression of canonical Notch target genes and *UNC5B* *in vitro*.** (A-B) EGTA induction (green lines) and CPE inhibition of EGTA (orange lines) of Notch target, *HEY2* (A), and *UNC5B* (B) in HUVECs (darker orange and green) and HRECs (lighter orange and green). (C-G) HUVECs were lentivirally transduced with RFP, ICN1, or ICN4. 24 hours after lentivirus infection, cells were harvested and analyzed by qPCR for *HES1* (C), *HEY1* (D), *NRARP* (E), and *RND1* (F) expression. (G) qPCR of *UNC5B* expression in pulmonary microvascular endothelial cells (PMVECs) transduced with ICN1 or ICN4 expression vectors and shCNT or shUNC5B, relative to RFP control. Multiple unpaired t-tests (A, B, G) and one-way ANOVA (C-F), presented as mean ± s.e.m. from at least 3 biological replicates per experiment.

**Supplemental Figure 3**

**
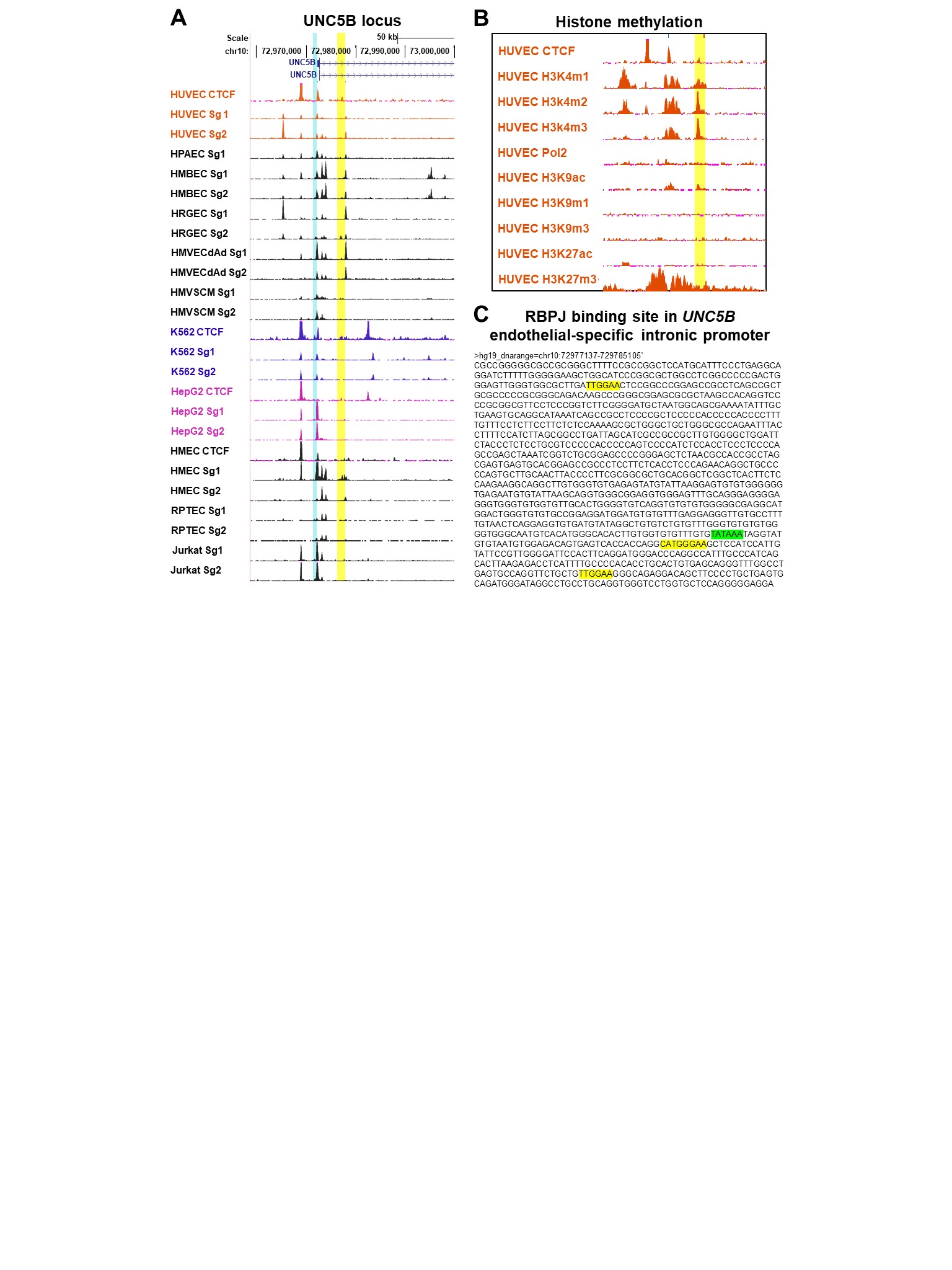
**

**Supplemental Figure 3. The UNC5B locus contains an active endothelial-specific promoter with RBPJ binding sites.** (A) Analysis of open chromatin regions (DNAse peaks) at the 5’ region of *UNC5B* (ENCODE Db at UCSC Genome Browser) shows two promoters. The 5’ promoter peak (blue highlight) shows open chromatin regions in several cell types, while the 3’ promoter peak located in intron 1 (yellow highlight) shows endothelial-specific chromatin peaks. (B) The endothelial-specific peak shows histone methylation patterns with higher occupancy of H3K4m3 over H3K4m1, indicating this region is a promoter. (C) DNA sequence of the endothelial-specific intronic promoter contains multiple consensus RBPJ binding domains (highlighted in yellow), suggesting that UNC5B is a direct Notch target. The transcriptional start site is highlighted in green.

**Supplemental Figure 4**


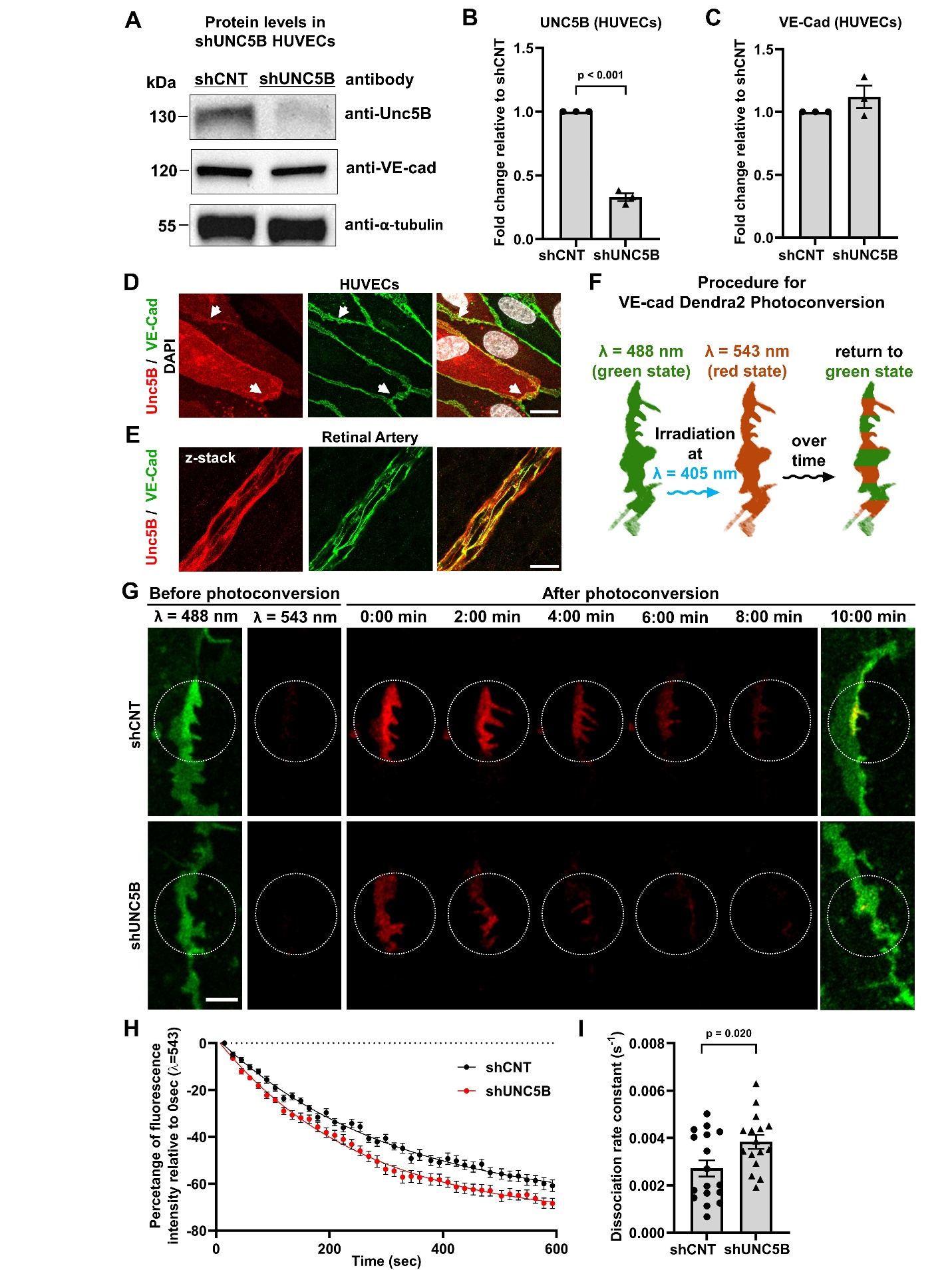


**Supplemental Figure 4. UNC5B regulates VE-Cadherin association with endothelial junctions.** (A) Western blot detection of UNC5B, VE-CADHERIN (VE-Cad), and α-TUBULIN (loading control) in lysates from shCNT and shUNC5B-treated HUVECs 72 hours after shRNA transduction. (B, C) Quantification for UNC5B and VE-Cad western blots, respectively, from 3 independent experiments. (D) IF image of HUVECs labeled for UNC5B (red) and for VE-Cad (green), with DAPI nuclei stain (gray). White arrow points to overlap between UNC5B expression at the VE-Cad positive junctions. (E) IF images of a postnatal day (5) retinal artery stained for Unc5B and VE-Cad. (F) Diagram of detection of VE-Cad disassociation rate via photoconversion of VE-Cad-Dendra2 fusion protein. (G) Time-lapse images of VE-Cad-Dendra2 emitting green fluorescence before photoconversion (λ = 488 nm) and red fluorescence (λ = 543 nm) after photoconversion within the irradiation zone (as indicated by the dashed circles) in shCNT and shUNC5B-treated HUVECs at basal time 0:00 (minutes:seconds) and for 8 minutes post-conversion. See also Supplemental Video 1. (H) Rate of loss of red fluorescent photoconverted VE-Cad within photoconversion zone in (G) as normalized to fluorescent intensity at time 0 hours. (I) VE-Cad disassociation rate constants from data in (G). n = 16-17 cell junctions from three independent experiments. Unpaired t-tests, presented as mean ± s.e.m. Scale bars, 15μm (D, E) and 3 μm (G).

**Supplemental Figure 5**

**
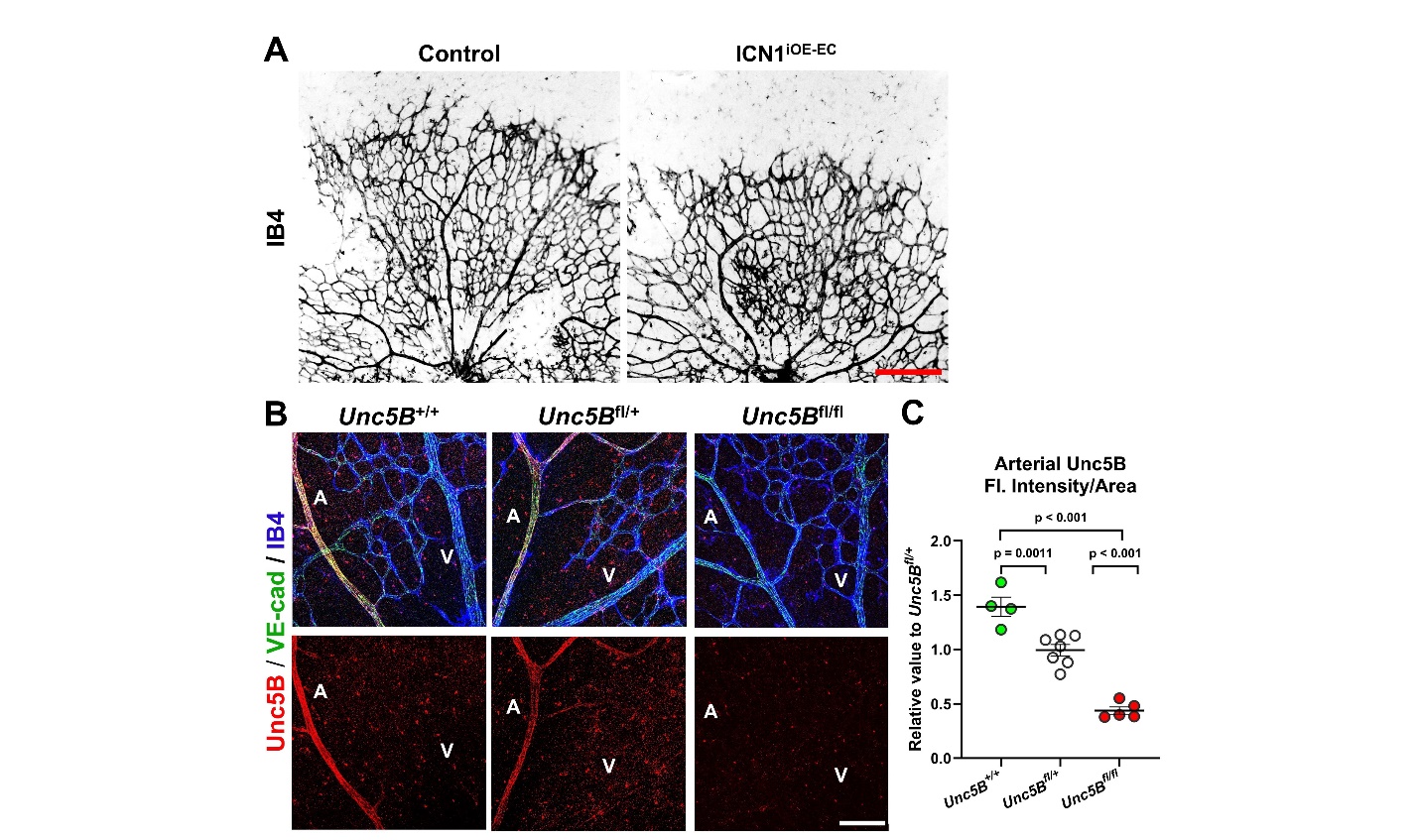
**

**Supplemental Figure 5. Validation of INC1 and Unc5B recombination in endothelial-cell specific ICN1^IOE-EC^ and Unc5B knockout mice.** (A) Representative images of Control and ICN1^IOE-EC^ retina showing predicted reduction of endothelial sprouting. (B) P5 retina stained for Unc5B (red) and the vasculature using VE-cadherin (VE-cad, green) and Isolectin B4 (IB4, blue) to evaluate Unc5B expression changes in *Cdh5-CreER^T2^* positive, tamoxifen-treated control (*Unc5B^+/+^*), heterozygous mutant (*Unc5B^fl/+^*) and homozygous Unc5B mutant (*Unc5B^fl/fl^*) mice. (C) Quantification of arterial Unc5B fluorescence (fl.) intensity per area relative to *Unc5b^fl/+^*. One-way ANOVA, presented as mean ± s,e.m. from at least three distinct animals per genotype. Scale bars, 175μm (A) and 100μm (B).

**Supplemental Figure 6**

**
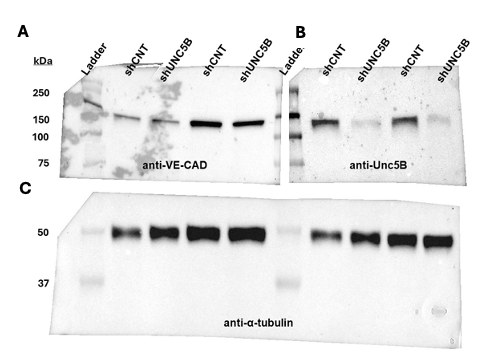
**

**Supplemental Figure 6: Uncropped images of Western blot.** (A-C) Uncropped Western blot of shCNT and shUNC5B treated HUVEC represented in Supplemental Figure 4.

**Supplemental Video 1**

**Supplemental Video 1: Detection of VE-Cad disassociation in shCNT and shUNC5B treated HUVEC.** Time-lapse video of shCNT (top panels) and shUNC5B (bottom panels) treated HUVEC expressing a full-length VE-Cad fusion to photoconvertible Dendra2. Left panels = 488 nm, unconverted VE-Cad-Dendra2. Middle panels = 543 nm converted VE-Cad Dendra2. Right panels = merge. Top righthand corner indicates time from photoconversion. Circle indicates region of photoconversion.

**Supplemental Table 1**. **qPCR primers.**

| Species | Gene | Forward Sequence (5’ 🡪 3’) | Reverse Sequence (5’ 🡪 3’) |
| --- | --- | --- | --- |
| Human | HES1 | CCTGTCATCCCCGTCTACAC | CACATGGAGTCCGCCGTAA |
| Human | HEY1 | ATCTGCTAAGCTAGAAAAAGCCG | GTGCGCGTCAAAGTAACCT |
| Human | HEY2 | GCCCGCCCTTGTCAGTATC | CCAGGGTCGGTAAGGTTTATTG |
| Human | NRARP | TCAACGTGAACTCGTTCGGG | ACTTCGCCTTGGTGATGAGAT |
| Human | RND1 | CTATCCAGAGACCTATGTGCC | CGGACATTATCGTAGTAGGGAG |
| Human | DLL4 | TGCAACTGCCCTTATGGCTTTGTG | ACAAGTTGTTCATGGCTTCCCTGC |
| Human | UNC5B | CTGGGACCTTATGCCTTCAA | CGCTTTGGTGGCAAAGTAAT |
| Human | NOTCH1 | GTCATCTCCGACTTCATCTACC | CGGAATCAGAGCGTGAGTAG |
| Human | NOTCH4 | AGATAAATGGGGGAAAACTGCG | CCTGGGCATCTTTATCGGCT |
| Human | GAPDH | GTCTCCTCTGACTTCAACAGCG | ACCACCCTGTTGCTGTAGCCAA |
| Human | ACTB | CGAGGCCCAGAGCAAGAGAG | CTCGTAGATGGGCACAGTGTG |

**Supplemental Table 2. List of antibodies used for immunofluorescence.**

| **Immunofluorescence:** | | | | |
| --- | --- | --- | --- | --- |
| Antibodies | Species | Source | Catalog | Dilution |
| ISOLECTIN-B4 | *Griffonia simplicifolia* | Vector Laboratories | B-1205 | 1:200 |
| anti-HA-tag | Rabbit | Abcam | ab9110 | 1:200 |
| anti-HA-tag | Rabbit | Cell Signaling Technology | 3724 | 1:200 |
| anti-VE-CADHERIN | Rat | BD Pharmingen | 555289 | 1:200 |
| Phalloidin | *Amanita phalloides* | Invitrogen | A-22287 | 1:500 |
| anti-UNC5B | Rabbit | Cell Signaling | 13851 | 1:250 |
| streptavidin Alexa 647 | Donkey | Invitrogen | S32357 | 1:700 |
| anti-rabbit Alexa 488 | Donkey | Invitrogen | A-21208 | 1:700 |
| anti-rat Alexa 647 | Donkey | Invitrogen | A-31573 | 1:700 |
